# Comparison of tick surveillance approaches: utilizing trailhead tick-check stations to support tick surveillance and community education

**DOI:** 10.3389/fpubh.2026.1919913

**Published:** 2026-10-07

**Authors:** Sabrina Gobran, Caroline Fagan, Lawson Dawe, Emma K. Harris, Christopher M. Roundy, Shelby Cagle, Nicole Kelp, Karla Saavedra-Rodriguez, Elizabeth Hemming-Schroeder

**Affiliations:** 1 Department of Microbiology, Immunology and Pathology, Colorado State University, Fort Collins, CO, United States; 2 Colorado Department of Public Health and Environment, Denver, CO, United States

**Keywords:** active tick surveillance, *Dermacentor*, KAP, One Health, passive tick surveillance, tick-borne disease, ticks, trail behavior

## Abstract

**Introduction:**

Rising tick-borne disease (TBD) cases and the geographical expansion of tick populations underscore the need for effective surveillance and public education regarding local risk. This study assessed the effectiveness of tick-check stations—self-service trailhead stations that provide tick and TBD information while allowing visitors to report trail use, tick encounters, and submit collected ticks—as a tool for tick surveillance and public education.

**Methods:**

To assess the effectiveness of tick-check stations for surveillance, we evaluated station engagement and compared tick density estimates, species composition, and sex- and life-stage distributions with those obtained through concurrent active surveillance. In addition, we compared submission numbers, and tick species, sex, and life stages to those collected through a mail-in submission system conducted by the Colorado Department of Public Health and Environment (CDPHE). To quantify feasibility, we estimated effort per tick and compared effort across simulated sampling scenarios. Finally, in-person knowledge, attitudes, and practices (KAP) interviews were conducted at trailheads to assess baseline tick KAP and to evaluate differences between sites with and without tick-check stations.

**Results:**

Engagement with tick-check stations was sustained throughout the study. Tick-check stations yielded 80 ticks, compared with 28 ticks collected through active surveillance and 28 ticks submitted through the CDPHE mail-in program. Tick-check station yield increased with underlying active-surveillance density (*β* = 0.378; 95% CI: 0.128–0.628; *p* = 0.003), while species composition, sex, and life-stage distributions were similar between the two methods. Tick-check stations and hybrid surveillance required less effort per tick than active surveillance when tick densities were low across all commute-time scenarios, with the greatest efficiency observed under high tick-station engagement. Participants who had read the tick-check station signage scored significantly higher in TBD knowledge than participants who had not (*p* = 0.006).

**Conclusion:**

Tick-check stations can provide tick density estimates comparable to active surveillance while requiring less effort in many scenarios, particularly in low-density settings. Our findings also highlight opportunities for targeted outreach to address gaps in TBD knowledge. As both a surveillance and educational tool, tick-check stations offer a sustainable approach for expanding tick monitoring in resource-limited settings.

## 1 Introduction

Since 2004, the number of reported tick-borne disease (TBD) cases in the United States (US) has been steadily rising (1). Concurrently, the geographical distribution of TBD incidence has expanded, mirroring the expansion of tick vector populations (2, 3). Multiple factors contribute to these trends, including shifts in habitat suitability induced by climate change, changes in land-use affecting host availability, increased awareness and reporting of TBDs, and the implementation of more tick surveillance systems and methods (4, 5). Given the persistence of climate change and environmental disturbances, ticks and their associated pathogens are likely to undergo continued shifts in distribution (6–8). Therefore, effective surveillance systems will be critical for the early detection of invasive vectors and emerging pathogens, ultimately supporting effective control and prevention of TBDs (4, 9–11).

While tick surveillance is essential for understanding and mitigating disease risk, its incorporation into routine public health programming is often constrained by cost, especially in areas with low TBD incidence (5). Active surveillance, typically involving systematic drag sampling over vegetation to collect questing ticks, provides geographically accurate, standardized estimates of tick presence and density (10, 12). However, this approach is resource-intensive, requiring significant time, labor, and logistical investment (13, 14). Alternatively, passive surveillance programs—typically relying on tick submissions from community members—are less cost-prohibitive, but yield less standardized and spatially accurate data, and are often biased by level of community awareness and engagement (9, 13, 15).

Current tick surveillance objectives, at both state and local agencies, function to detect the presence and abundance of ticks and evaluate tick-borne pathogens to understand public health risks (5). Most surveyed agencies conducted tick surveillance through passive programs (typically via a mail-in system), or by irregular active surveillance activities. However, up to 35% of these agencies reported being unable to carry out the main objectives of their tick surveillance programs, primarily due to lack of funding and resources (5). Although passive surveillance programs may be limited in standardization and spatial resolution, this approach can still help address certain public health objectives when systematic active surveillance is not feasible. Passive surveillance may even prove superior to active sampling in measuring realized tick-human encounters, a dimension not typically captured by most active surveillance methodologies (16).

Given the limitations of traditional passive surveillance approaches, namely, the underutilization of these programs by the public, that data may be biased and lack standardization, and the high costs and labor demands of active surveillance, we developed an alternative method based on implementation of trailhead tick-check stations. These stations provide a low-effort, accessible platform for academic researchers and public health agencies to collect ticks by engaging community members to directly submit ticks encountered during outdoor activities at conveniently located submission stations. This approach leverages human-tick interactions while minimizing logistical burden. Trailheads were targeted for placement of tick-check stations, as tick encounters in Colorado are most likely to occur while recreating in natural areas (17).

Tick-check stations may also provide an opportunity for targeted public health education. Colorado hikers and outdoor recreationalists are at a higher risk of encountering ticks and tick-borne pathogens (18, 19), and placement of educational materials at trailheads allows prevention messaging to reach individuals during potentially high-risk activities. Previous studies of tick knowledge, attitudes, and practices (KAP) have primarily focused on individuals with high occupational exposure to ticks (e.g., Forest Service employees, farmers, veterinarians) (20–22) or individuals with recreational exposure (23, 24). The KAP framework provides a structured approach to examine whether changes in knowledge and risk perception translate into sustained protective practices (25, 26). Although outdoor workers often reported receiving inadequate information on tick bite prevention (21, 27, 28), these groups may be more accessible for intervention through structured training and resource provision. Recreational users generally lack these structured opportunities for education, and their risk varies widely with the frequency, duration, and type of activity (23). Trailheads may therefore provide a useful setting for targeted prevention messaging.

To evaluate tick-check stations as both a surveillance and public health education tool, we installed tick-check stations at four locations in northern Colorado during spring–summer 2024, coinciding with peak host-seeking activity season of *Dermacentor andersoni* (Rocky Mountain wood tick) and *Dermacentor variabilis* (American dog tick), and conducted in-person KAP interviews at trailheads with and without tick-check stations. Our objectives were to (1) evaluate station engagement and determine whether tick-check station submissions provided geographically relevant estimates of tick abundance and composition comparable to active surveillance and the mail-in passive surveillance program operated by the Colorado Department of Public Health and Environment (CDPHE), (2) compare the resource requirements and efficiency of tick-check stations with active and combined surveillance approaches, and (3) assess whether exposure to tick-check station educational signage was associated with differences in tick- and TBD-related KAP. We hypothesized that trailhead tick-check stations would provide surveillance estimates comparable to active surveillance while requiring fewer resources and that informational signage at the stations would be associated with greater tick and TBD knowledge.

## 2 Materials and methods

### 2.1 Overview of study design

We evaluated tick-check stations, which combined educational signage with a self-service system for reporting trail activity and tick encounters and submitting collected ticks (Figure 1), using three complementary components: surveillance performance, operational efficiency, and public education. Surveillance performance was assessed by comparing tick-check station submissions, a passive surveillance approach, with researcher-conducted drag sampling (active surveillance) at four northern Colorado sites and with submissions to the CDPHE mail-in tick submission program. Operational efficiency was evaluated by comparing effort per tick among tick-check stations, active surveillance, and a combined tick-station and active-surveillance approach. Educational outcomes were assessed using in-person KAP interviews conducted at trailheads with and without tick-check stations.

**Figure 1:**
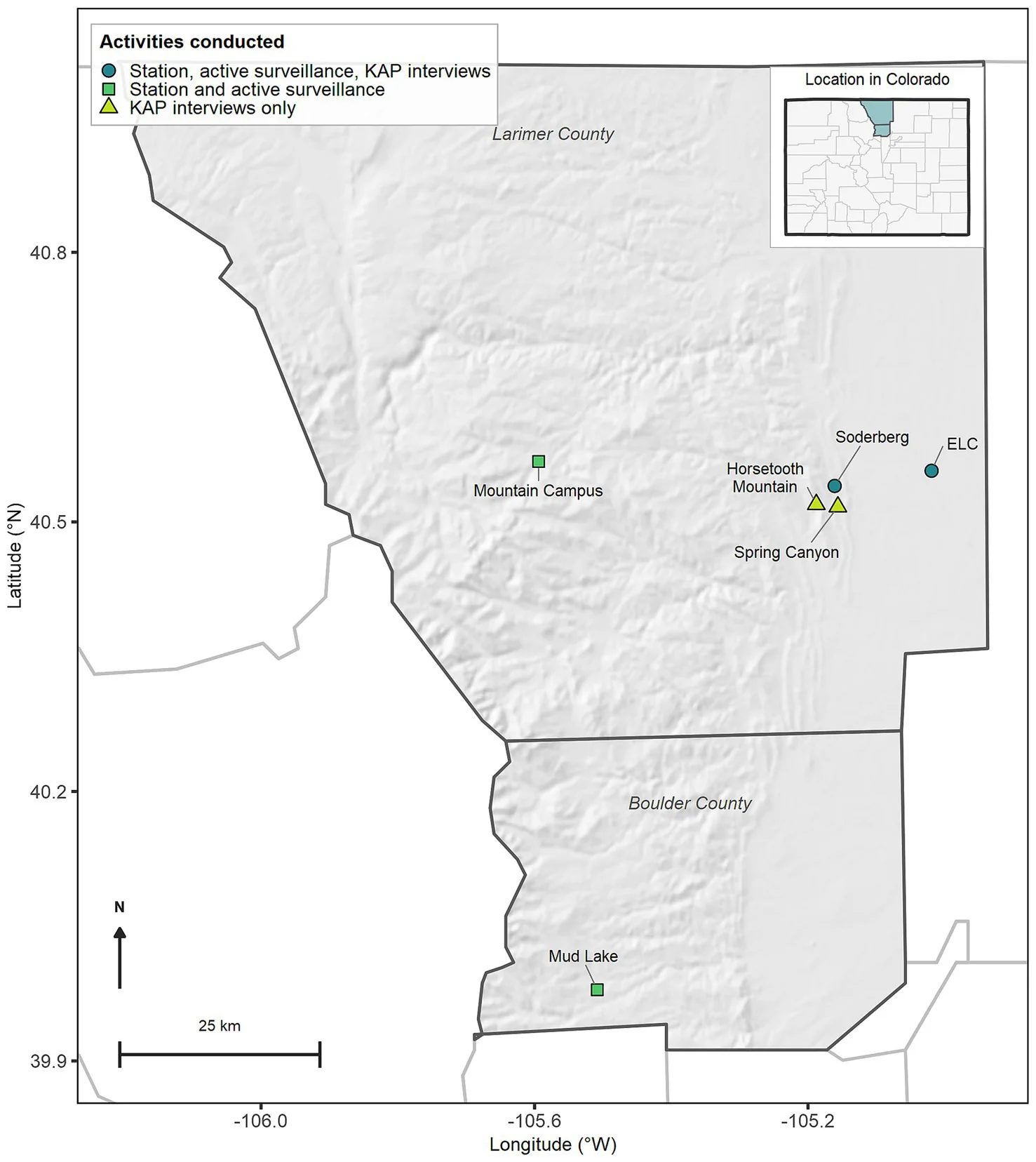
Study site locations in Larimer and Boulder counties, Colorado. Color and shape of site points indicate the research activities conducted at each. Coordinates and site characteristics are provided in Supplementary Table S1. Relief shading is derived from a digital elevation model (AWS Terrain Tiles via the *elevatr* package), illuminated from the north-west.

### 2.2 Study location

Study activities took place at six locations across Boulder and Larimer counties, Colorado (Figure 2). The study sites fall within the Southern Rockies, a montane ecosystem where elevational gradients drive vegetation and climate (29). Sites included lower-elevation foothill locations (ELC, Soderberg, Horsetooth Mountain, and Spring Canyon Trail; < 2000 m) and higher-elevation montane locations (Mud Lake and CSU Mountain Campus; > 2000 m), selected to encompass habitats associated with *D. variabilis* and *D. andersoni*, respectively (17, 30–32). Lower-elevation sites included grassland and foothill shrubland, whereas higher-elevation sites included lodgepole and ponderosa pine forest; site-specific elevation and habitat characteristics are summarized in Supplementary Table S1. Active surveillance, tick-check station passive surveillance, and KAP surveys were conducted at ELC and Soderberg. Active surveillance and tick-check station passive surveillance were conducted at Mud Lake and CSU Mountain Campus. KAP surveys alone were conducted at Horsetooth Mountain and Spring Canyon Trail. All sites were located on publicly accessible trails, except for the CSU Mountain Campus. While the CSU Mountain Campus is not a public natural area or trail system, this campus is populated with students in the summer and has a high tick encounter rate (33). The tick-check station was placed in a central location where it was accessible to the students.

**Figure 2:**
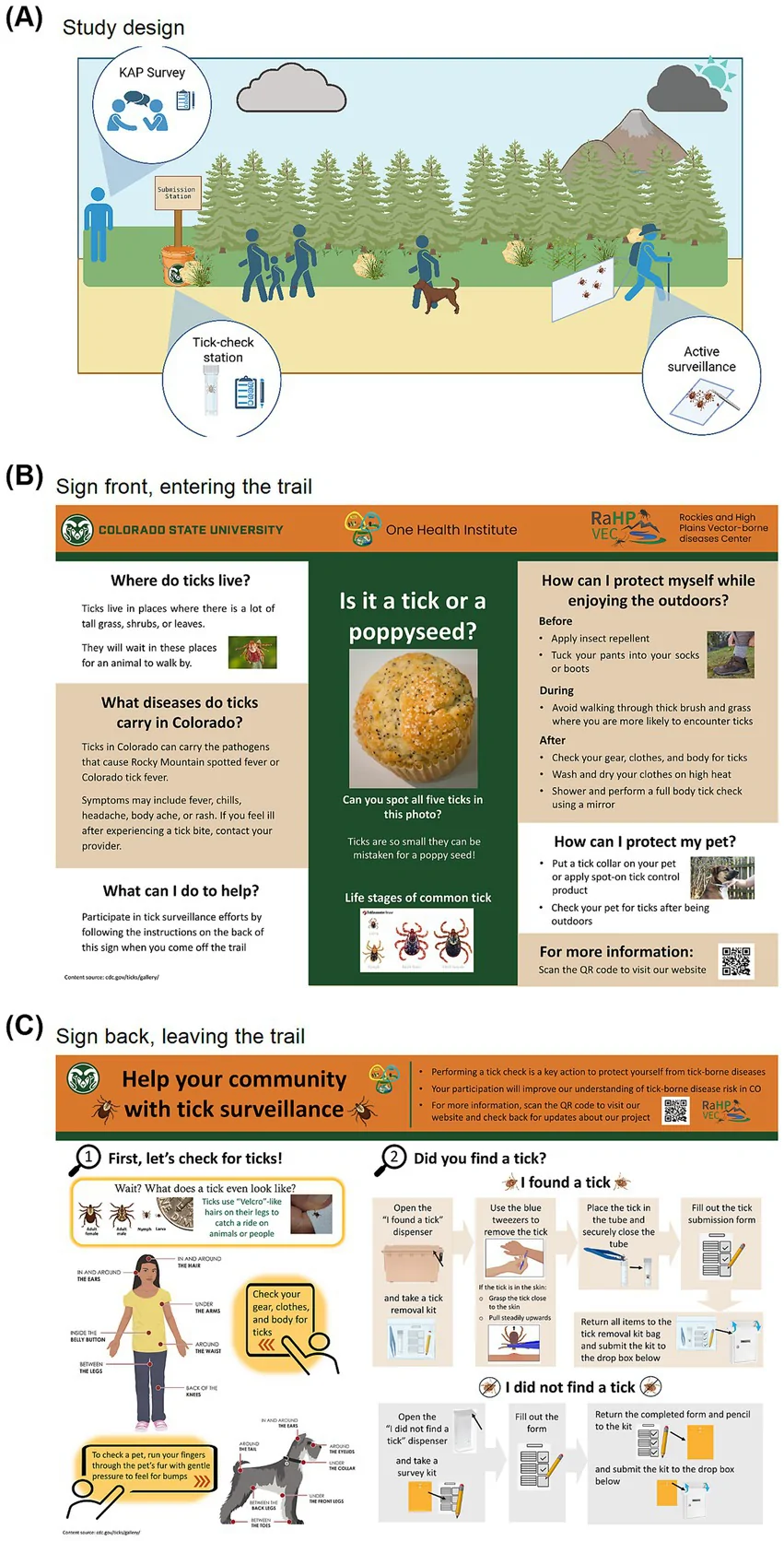
Study design and tick-check station signage. **(A)** Trail users encountered a tick-check station at the trailhead, performed a tick check when leaving the trail, and submitted any ticks found together with a short survey; active surveillance was conducted at the same sites by drag sampling, and trail users were separately interviewed in-person about their tick knowledge, attitudes, and practices. **(B)** Front of the station sign, seen by trail users on their way to the trail, giving information on where ticks occur, the diseases they transmit in Colorado, and how to prevent tick bites. **(C)** Back of the station sign, seen on returning from the trail, giving illustrated instructions for performing a tick check and for submitting a tick or a completed survey form to the station.

### 2.3 Tick-check stations

Tick-check stations were designed to be placed at trailheads, allowing hikers to view information when entering and exiting the trail (Figure 1). Stations were operated from April through August 2024, with deployment periods varying by site: ELC, April 21–August 4; CSU Mountain Campus, May 22–July 16; Mud Lake, May 24–July 28; and Soderberg, May 22–July 31. At the CSU Mountain Campus, the tick-check station was centrally located to maximize participation. The front of the sign, which was viewed by hikers when approaching the trail, included information about ticks and TBDs in Colorado. The rear of the sign, which was viewed by hikers when exiting the trail, included information about how to conduct a tick check and participate in the tick submission program. If hikers found a tick, they were directed to retrieve a tick submission kit, which contained a survey form, pencil, submission tube, disposable forceps, and disinfecting wipe. Submission tubes contained no preservative. If hikers did not find a tick, they were directed to an alternative dispenser which contained kits that only included a survey form and a pencil. The survey form included in both kits was identical; the only difference between the kits were the additional contents supplied for tick handling. Completed surveys and ticks were submitted to a locked drop box attached to the station. Stations were visited every 1–2 weeks to replenish supplies and collect submissions, and submitted ticks were recovered alive from the stations. Materials and instructions for building the tick-check stations and survey questions are provided in the Supplementary Methods S1, S2.

### 2.4 CDPHE mail-in tick submission program

Ticks were submitted to CDPHE by members of the public and veterinarians; Larimer County submissions included in this study were received between April 13 and June 18, 2024. Prior to submission, contributors completed a standardized form capturing key metadata, including the county of collection, specific geographic location (e.g., trail, park, or address), host association (if applicable), and host type. Submitted ticks were double bagged in zip top bags and mailed to CDPHE for processing. Results were reported back to the submitter following identification.

### 2.5 Active surveillance

Active surveillance was conducted from April through August 2024, with sampling periods varying by site: ELC, April 15–July 17; Soderberg, April 25–July 24; CSU Mountain Campus, May 16–August 14; and Mud Lake, May 21–July 22. Sites were sampled at 1–2 week intervals, with sampling dates varying among sites. We used a standard dragging approach for active surveillance, which involves dragging a 1-m^2^ white cloth along the forest floor and removing any attached ticks (34). Sampling was conducted along 20-meter transects parallel to the trail edge every 1–2 weeks. Starting points for each transect were randomly selected along the trail during each visit. Transects markers were placed on either side of the trail at 0 m, 5 m, and 10 m from the trail edge, where accessible. Sampling was conducted on each side of the transect markers. Drags were inspected every 5 m, and any attached ticks were removed with forceps and placed into uniquely identified vials. Data was entered electronically using the Open Data Kit (ODK) Collect mobile data collection platform.^1^

### 2.6 Tick identification

Ticks collected through tick-check stations and active surveillance were transported to CSU and kept alive at 4 °C until they could be identified. Tick species, life stage, sex, and engorgement status were identified by microscopy and standard dichotomous keys (35, 36). Tick morphology was also compared with the newly described *Dermacentor similis* (37). After identification, ticks were preserved at −80 °C for downstream RNA-based pathogen testing and potential virus isolation. Ticks submitted through the CDPHE mail-in program were identified separately by CDPHE state entomologists to species, life stage, sex, and engorgement status using microscopy and standard dichotomous keys.

### 2.7 KAP interviews

We designed a nine question, in-person, verbal survey consisting of Likert scale, Y/N, multiple choice, and open response questions to evaluate hiker KAP (Supplementary Methods S3, S4) based on the survey design of Cuadera et al. (38). Interviews were conducted in-person from late June to early September 2024. Interview respondents were approached and gave verbal consent to participate in the study as they were leaving the trail. Researchers read interview questions to the participant(s), and input participant verbal responses into the ODK platform. Responses were recorded without identifying information, although demographic information was collected (Supplementary Table S2).

To assess knowledge, interview participants were asked to correctly identify (1) proper tick removal technique, (2) ticks from a series of images, and (3) TBDs endemic to Colorado. To assess attitudes, interview participants were asked to rate their concern for tick bites and TBDs on a scale of 1–5. To assess practices, interview participants were asked whether they take any precautions to prevent tick bites. Survey responses were coded into analysis variables according to predefined criteria summarized in Supplementary Table S3.

### 2.8 Statistical analysis

All statistical analyses were conducted in R (version 4.2.0). Every test reported in the manuscript, with its degrees of freedom, effect size, confidence interval and *p* value, is listed in Supplementary Table S4. Trail-use characteristics were summarized by study sites using the *gtsummary* package. Differences among sites in categorical variables were evaluated using Pearson’s chi-squared tests, or Fisher’s exact tests when expected cell counts were below five, with Cramer’s V as the effect size. Continuous and ordinal variables were compared using Kruskal–Wallis tests. We tested whether the likelihood of a tick encounter differed with trail-use behaviors and environmental conditions. Tick encounter was recorded as a binary variable based on whether a participant reported finding at least one tick during their tick check. Associations with time of day, walking through grass, bringing a pet, and weather conditions were evaluated using Fisher’s exact tests, while hike duration was compared between participants who did and did not encounter a tick using Kruskal-Wallis tests. Because tick encounter differed markedly among sites, each association was then re-estimated in a logistic regression adjusting for site.

To understand whether engagement with the tick stations was sustained over the season, we modeled the number of surveys submitted per week separately for each site. Weekly survey count was the dependent variable and week was entered as a linear term. Counts were modeled with a negative-binomial generalized linear model fit with *glmmTMB* and a log link. To evaluate whether tick composition differed among collection methods, we compared species composition and life-stage/sex composition among active surveillance, tick-check stations, and the CDPHE mail-in program using Fisher’s exact tests. *p* values were adjusted by the Benjamini-Hochberg correction method to account for multiple comparisons.

To compare tick abundance measured through tick station surveillance and active surveillance, we aggregated data into non-overlapping 14-day site-specific periods and calculated passive tick yield as ticks submitted per tick-check survey and observed active-surveillance density as ticks collected per 1,000 m^2^ sampled. To account for unequal active sampling effort and differences among sites, we estimated underlying active-surveillance density using a hierarchical Gamma-Poisson model. Active tick counts were modeled using a negative-binomial mixed-effects model using a log link, an offset for sampling effort, and a random intercept for site. The fitted model was used to derive site-specific Gamma distributions for latent active-surveillance density, which were updated for each paired site-period using its observed active tick count and sampling effort. The median of the resulting distribution was used as the latent density estimate. We then tested whether passive tick yield increased with underlying active-surveillance density using a Poisson generalized linear mixed model. Passive tick count was the response, log(1 + median latent active-surveillance density) was the predictor, the log-transformed number of submitted surveys was included as an offset, and site included as a random intercept. Model adequacy was evaluated using DHARMa residual diagnostics (39). To assess whether the relationship with latent active-surveillance density reflected more than underlying differences among sites, we compared the final model with a reduced model containing the survey-effort offset and site random intercept but excluding latent active-surveillance density, using a likelihood-ratio test and AIC. As a robustness analysis, we refit the model after excluding the paired site-period with the highest observed active-surveillance density.

We used the fitted passive-surveillance model to compare the efficiency of active surveillance, tick stations, and a hybrid strategy combining both approaches over standardized two-month surveillance periods. Scenarios assumed underlying tick densities of 0.2, 1, 2, and 4 ticks per 1,000 m^2^ (selected to approximate densities represented among 14-day period/sites) and one-way travel times of 30, 60, or 120 min. Tick stations were assumed to require two site visits per month and 4 h per month for preparation and data management. Passive engagement was evaluated at 15 surveys per month (low engagement) and 30 surveys per month (high engagement). These engagement values were based on observed engagement at our four sites where we received an average of 15.0 tick-check surveys per month at Mud Lake, 20.0 at Soderberg, 31.1 at ELC, and 32.7 at CSU Mountain Campus. These values represent total surveys, including those where a tick was not found during the tick check. For active surveillance, we assumed that 1,000 m^2^ would be sampled per visit and that it would take 150 min to sample 1,000 m^2^. For each density and engagement scenario, we predicted expected tick-station yield from the fitted passive-surveillance mixed model. Predictions were generated separately for each study site using its fitted random intercept and then averaged across sites on the response scale. Holding this fitted relationship fixed, we simulated 5,000 two-month surveillance periods to represent variation in realized tick collections. Passive tick counts were drawn from Poisson distributions defined by the model-predicted station yield, whereas active tick counts were drawn from Poisson distributions defined by the specified underlying density and 4,000 m of sampling effort. For the hybrid strategy, simulated passive and active collections were combined, with active sampling assumed to occur during the same two monthly visits required to service the tick station and therefore requiring no additional travel. Surveillance efficiency was expressed as total minutes of effort per expected tick collected and summarized using the median and 95% simulation interval.

We grouped KAP interview participants into control or intervention groups by whether they reported reading the informational signage. Because interviews were conducted as participants left the trail, knowledge outcomes may reflect exposure to the signage, whereas attitudes and habitual practices were largely determined beforehand and participants self-selected into reading the sign; group comparisons for attitudes and practices are therefore reported as baseline comparability rather than as effects of the intervention. Open-ended responses were coded by matching pre-specified terms against the verbatim text, case-insensitively and allowing for the spelling variants present in the data; a response naming several items was counted under each. The coding criteria for every question are given in Supplementary Table S3.

For disease knowledge, participants were assigned a score from 0–2. If they were unable to name a TBD or only named a TBD non-endemic to Colorado (e.g., Lyme disease, anaplasmosis), they were assigned a 0. If they named both an endemic and non-endemic TBD (e.g., Lyme disease and RMSF), they were assigned a 1. If they named only TBDs endemic to Colorado (RMSF or CTF) they were assigned a 2 (40–43). Tick removal responses were coded for four independent elements: (1) naming a removal instrument (e.g., tweezers), (2) indicating where to grasp the tick (i.e., by the head), (3) describing how to pull (e.g., steadily, without twisting), and (4) describing an unsafe/hazardous method (e.g., using substance or heat to make the tick detach, twisting the tick). Each element is reported separately, and the four are combined into a binary removal knowledge score: participants scored 1 if they described at least one of the three correct elements (#1–3) and no unsafe method (#4), and 0 otherwise (i.e., they described an unsafe method, described none of the correct elements, or gave no answer). Correct image identification was scored from 0–1 and required selecting exactly the two tick images with no non-tick images to receive a 1. Knowledge outcomes were compared by signage exposure (read vs. did not read) using Wilcoxon rank sum tests for disease knowledge and Pearson’s chi-squared tests for tick removal and identification.

Open ended prevention practices were broadly classified as preventative or post-exposure behaviors. Preventative behaviors were subclassified as related to clothing (e.g., tucking pants into socks), repellent (e.g., applying DEET), and trail behavior (e.g., staying on designated trails). Post-exposure behaviors were subclassified as performing tick checks, taking a shower, or washing clothes after hiking. Individual prevention practices were tested using Pearson’s chi-squared test where every expected cell count was at least five and Fisher’s exact test otherwise. Attitudes measured on Likert scales were compared by signage exposure using Fisher’s exact tests on the response distribution. The association between each attitude and each reported practice within the sample was quantified with Kendall’s rank correlation, omitting practices reported by fewer than five participants. Knowledge and practice outcomes were also examined in relation to residence history in a state the CDC classifies as high incidence for Lyme disease (44). We defined high incidence Lyme disease states as: Connecticut, Delaware, District of Columbia, Maine, Maryland, Massachusetts, Minnesota, New Hampshire, New Jersey, New York, Pennsylvania, Rhode Island, Vermont, Virginia, West Virginia, Wisconsin.

### 2.9 Ethical approval

The KAP interview questionnaire, and its protocol, were determined to be exempt under category 2i by the Colorado State University Institutional Review Board (IRB; Protocol No. 5655).

## 3 Results

Across all surveillance approaches, 136 ticks were collected: 28 through active surveillance, 28 through the CDPHE mail-in program, and 80 through tick-check stations. Active surveillance yielded 27 *D. andersoni* and one *D. variabilis*, comprising 19 adult females, seven adult males, and two nymphs. Of these, 26 ticks were collected at CSU Mountain Campus, one at ELC, and one at Mud Lake. Tick-check stations yielded 74 *D. andersoni* and eight *D. variabilis*, comprising 51 adult females and 31 adult males before exclusions (see below). Most tick-check station specimens were collected at CSU Mountain Campus (*n* = 71), followed by ELC (*n* = 8), and Mud Lake (*n* = 1). Two specimens submitted at Soderberg were excluded from analysis: one was acquired away from the trail and one was not a tick. The CDPHE mail-in program yielded 13 *D. andersoni* and 15 *D. variabilis*, including 23 adult females and five adult males. No tick collected from any approach was engorged.

### 3.1 Trail behavior and engagement

Overall, 257 tick-check surveys were submitted across the four tick-check stations between April and August 2024 (Table 1). The highest number of tick-check surveys were collected from the ELC (*n* = 113), and the fewest were collected from Mud Lake (*n* = 32). Among respondents reporting a survey submission time, 76/249 (31%) submitted in the morning, 108/249 (43%) in the afternoon, and 65/249 (26%) in the evening. Overall, 120/254 (47%) respondents reported walking through ankle-length or taller grass or brush, and 25/243 (10%) reported recreating with a pet. Sunny conditions were most frequently reported (171/252, 68%), followed by partly cloudy (97/252, 38%), windy (41/252, 16%), cloudy (27/252, 11%), and rainy (17/252, 6.7%); respondents could select more than one weather condition. Sixty of 257 surveys (23%) reported finding at least one tick. Site-specific response distributions and hike durations are provided in Table 1.

**Table 1:** Trail-use characteristics reported on tick-check station survey forms at four northern Colorado sites, April–August 2024.

Characteristic	ELC	CSU Mountain Campus	Mud Lake	Soderberg	*p*-value
Time of submission					<0.001
Morning	31/109 (28%)	11/54 (20%)	6/31 (19%)	28/55 (51%)	
Afternoon	47/109 (43%)	18/54 (33%)	21/31 (68%)	22/55 (40%)	
Evening	31/109 (28%)	25/54 (46%)	4/31 (13%)	5/55 (9.1%)	
Time on trail (hours)	1.25 (1.00, 1.50)	1.78 (0.23, 3.00)	1.19 (0.75, 1.50)	1.87 (1.00, 2.12)	<0.001
Distance hiked (miles)	1.60 (1.00, 2.00)	2.27 (0.25, 3.50)	1.64 (1.00, 2.20)	4.13 (2.50, 5.00)	<0.001
Walked through ankle-length or taller brush	51/111 (46%)	36/54 (67%)	6/32 (19%)	27/57 (47%)	<0.001
Brought a pet onto the trail	1/104 (1.0%)	0/53 (0.0%)	9/31 (29%)	15/55 (27%)	<0.001
Weather conditions					
Sunny	72/111 (65%)	34/52 (65%)	20/32 (63%)	45/57 (79%)	0.237
Partly Cloudy	47/111 (42%)	26/52 (50%)	11/32 (34%)	13/57 (23%)	0.021
Cloudy	12/111 (11%)	5/52 (9.6%)	4/32 (13%)	6/57 (11%)	0.972
Windy	11/111 (9.9%)	14/52 (27%)	5/32 (16%)	11/57 (19%)	0.046
Rainy	8/111 (7.2%)	5/52 (9.6%)	0/32 (0.0%)	4/57 (7.0%)	0.375
Tick encounters					<0.001
Surveys reporting a tick	8/113 (7.1%)	48/54 (89%)	2/32 (6.3%)	2/58 (3.4%)	
Surveys with > = 1 confirmed tick	8/113 (7.1%)	46/54 (85%)	1/32 (3.1%)	0/58 (0.0%)	
Total confirmed ticks submitted	8	71	1	0	

Categorical values are *n*/*N* (%), where *N* is the number of surveys with a non-missing response for that item, so denominators differ among rows. Continuous values are mean (Q1, Q3). *p* values compare sites: Pearson’s chi-squared, or Fisher’s exact test where expected counts were below 5, and Kruskal-Wallis for continuous variables. Pets were prohibited at the ELC and the Mountain Campus, so that comparison reflects site access policy rather than visitor behavior. *n*/*N* (%); mean (Q1, Q3). *N* is the number of surveys with a non-missing response for that item, so denominators differ among rows. Weather was a select-all-that-apply item.

Time of survey submission varied significantly by site (*V* = 0.26; *p* < 0.001), with afternoon most common at the ELC (47/109, 43%) and Mud Lake (21/31, 68%). The evening was most common at the CSU Mountain Campus (25/54, 46%), while the morning was the most common at Soderberg (28/55, 51%). Hike distance and duration varied significantly by site (ε^2^ = 0.24, *p* < 0.001; ε^2^ = 0.08, *p* < 0.001, respectively), with the longest average duration (1.87 h) and greatest mean distance (4.13 miles) at Soderberg. Mud Lake had the shortest average duration (1.19 h) and ELC the shortest distance (1.60 miles). The proportion of respondents reporting that they walked through ankle-length brush varied by site (*V* = 0.27; *p* < 0.001), and was highest at CSU Mountain Campus (36/54, 67%) and lowest at Mud Lake (6/32, 19%). The proportion of respondents who recreated with pets also varied among sites (*V* = 0.43; *p* < 0.001), but pets are prohibited at the ELC and CSU Mountain Campus, so this comparison reflects site access policy. Across all four sites, “sunny” weather was the most reported. Among the five conditions, partly cloudy (*V* = 0.20; *p* = 0.021) and windy (*V* = 0.18; *p* = 0.046) differed among sites; sunny, cloudy and rainy did not (*p* > 0.05). Tick encounters differed significantly among sites (*p* < 0.001): 48/54 (89%) of CSU Mountain Campus surveys reported a tick, against 8/113 (7.1%) at the ELC, 2/32 (6.3%) at Mud Lake, and 2/58 (3.4%) at Soderberg.

Associations between each trail-use characteristic and the likelihood of reporting a tick were estimated first without adjustment and then in binomial generalized linear mixed models with a random intercept for site (Supplementary Table S5). Unadjusted, three characteristics were associated with finding a tick. Evening submissions were more likely to report a tick than morning submissions (25/65, 38% versus 12/76, 16%; crude OR 3.33, 95% CI: 1.53–7.58); respondents who walked through ankle-length or taller brush were more likely to report a tick than those who did not (34/120, 28%; crude OR 2.39, 95% CI: 1.29–4.55); and windy conditions were associated with a tick report (14/41, 34%; crude OR 2.44, 95% CI: 1.15–5.05). However, none of these associations persisted after accounting for site.

We assessed community engagement with the tick-check stations over time at each of the four sites (Figure 3). Engagement was determined by the number of tick-check survey submissions per week. The first tick-check station was placed at the ELC during the week of April 15, 2024, while the other three stations were set up during the week of May 20, 2024. All stations received submissions throughout the study duration. There was no significant difference in tick-check surveys submitted per week for the Mud Lake station (RR = 0.90; 95% CI: 0.81–1.13; *p* = 0.566). There was a significant decrease in submissions per week at the ELC, CSU Mountain Campus, and Soderberg (*p* < 0.001).

**Figure 3:**
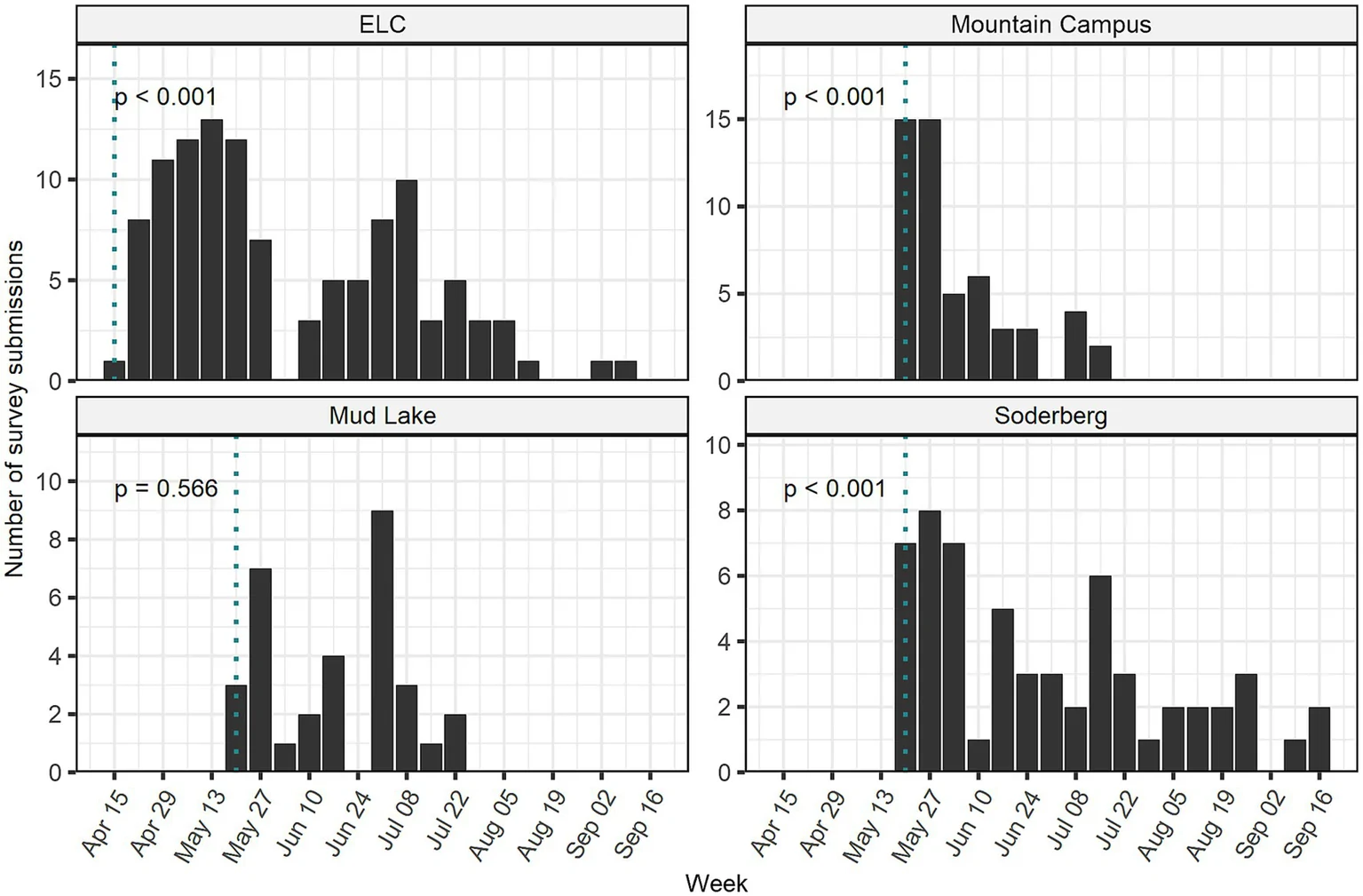
Tick-check station survey submissions per week at each of the four station sites, Colorado, April–August 2024. The dotted line indicates the week that the station was installed. *p* values test a linear trend in weekly submission counts, from negative-binomial generalized linear models fitted separately by site.

### 3.2 Comparison of tick-check station and the CDPHE tick mail-in program

The CDPHE tick mail-in program is a statewide passive surveillance initiative that is divided amongst counties in Colorado (45). Since our study was restricted to northern Colorado, we compared the results from our tick-check stations to mail-in data from Larimer County only. Mud Lake is located in Boulder County and was thus excluded from these analyses. Although the CDPHE mail-in system was available year-round, and our tick-check stations were only available during the summer months, the tick submission date range was comparable between the two surveillance programs. From the date of the first submission to the date of the final submission for the mail-in system was 66 days and for our tick-check stations was 79 days. Therefore, even though our program was available to the community for a shorter period, the duration of engagement was comparable, indicating that the availability of the tick-check stations coincided with the period of relevant tick encounter risk. Further, the mail-in system captured more geographic variation compared to the tick-check stations (Supplementary Figure S1). Since our tick-check stations were only available at a limited number of specific sites, we were only able to capture a small amount of geographic variation.

Overall, there was a higher number of ticks submitted to our tick-check stations than through the mail-in program. Within Larimer County alone, we received 79 ticks from our tick-check stations, while 28 ticks were submitted to the CDPHE mail-in system. There was a more even distribution of tick species, *D. andersoni* and *D. variabilis*, submitted through the mail-in system than the tick-check stations (Figure 4). The variability in tick species collected through the mail-in and the tick-check station was significant (OR = 11.48; 95% CI: 3.6–40.55; *p* < 0.001) with a greater proportion of *D. variabilis* ticks submitted through the mail-in system than through the tick-check stations. In contrast, there was no significant difference in adult sex composition between the tick check-stations and mail-in program, with adult females comprising the predominant stage for both (OR = 0.34; 95% CI: 0.09–1.05 *p* = 0.119). No nymphs or larvae were collected by either passive surveillance method.

**Figure 4:**
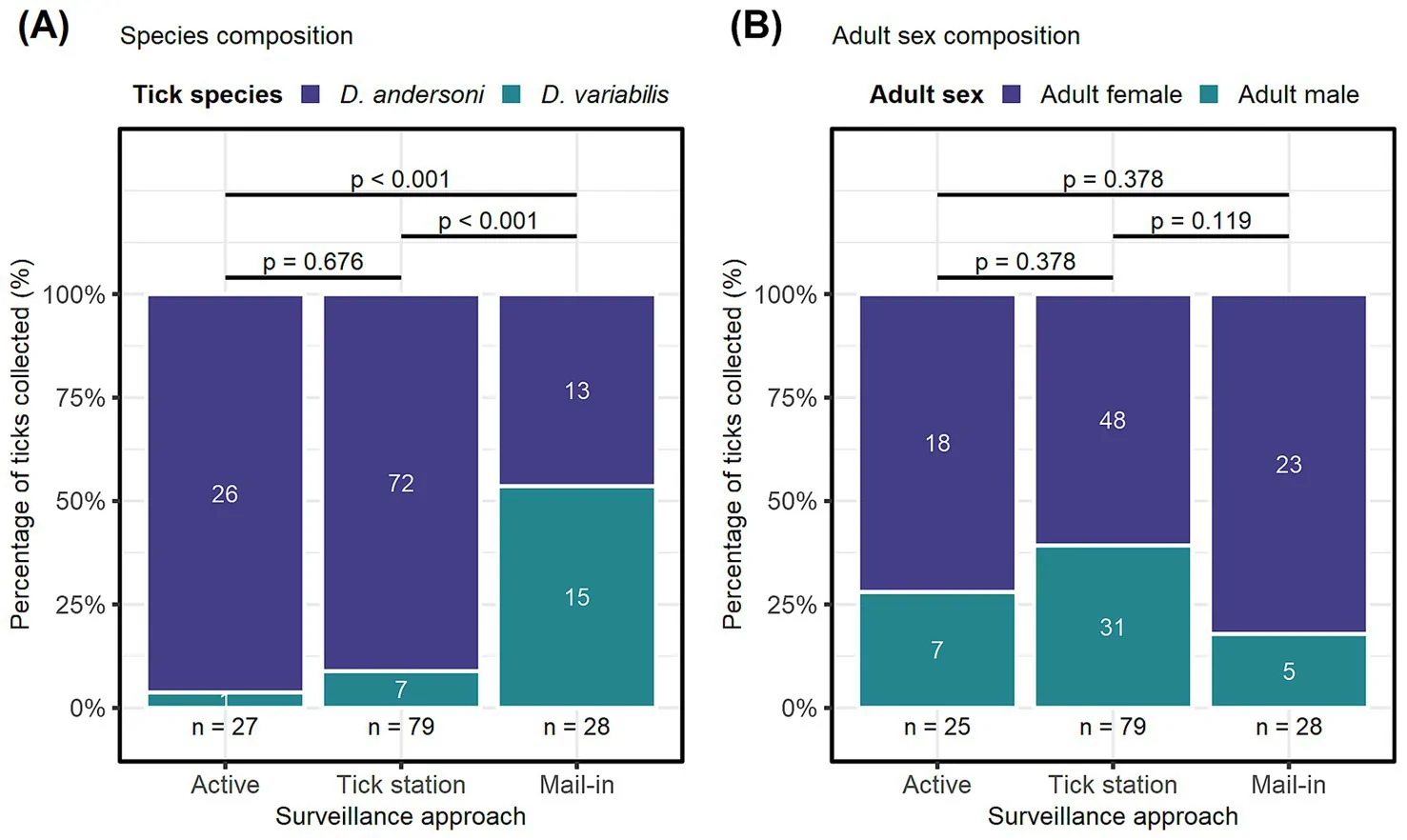
Tick species and adult sex composition by surveillance approach, Larimer County, Colorado, April–August 2024. Stacked bars show the percentage composition of ticks collected by active surveillance (*n* = 27), tick-check stations (*n* = 79), and the CDPHE mail-in program (*n* = 28). **(A)** Species composition of *D. andersoni* and *D. variabilis*. **(B)** Adult sex composition. Pairwise differences among surveillance methods were evaluated using Fisher’s exact tests; *p* values are shown above the corresponding comparisons. Mud Lake is excluded from the figure because the CDPHE mail-in data used only included Larimer County. Two nymphs were collected by active surveillance and none by either passive approach; immature stages are reported descriptively. Brackets show pairwise Fisher’s exact tests with Benjamini-Hochberg adjustment.

### 3.3 Comparison of tick-check station and active surveillance

There was not a significant difference between tick species collected through active surveillance and tick-check station (OR = 2.51; 95% CI: 0.30–118.18; *p* = 0.676; Figure 4). Among adult ticks, sex composition did not significantly differ between active surveillance and tick-check stations (OR = 1.65; 95% CI: 0.57–5.25 *p* = 0.378). Adult females were the predominant sex collected by each approach. Two nymphs were collected through active surveillance, whereas no nymphs were collected through either passive surveillance approach. We next compared tick density over time by active surveillance and tick-check station at the three sites where ticks were collected (Figure 5). No ticks were collected from either surveillance method at the Soderberg site, so it was excluded from further analysis. For our tick-check stations, density was calculated as the number of ticks collected per tick-check survey submitted. For active surveillance, density was calculated as the number of ticks collected per 1,000 m^2^ sampled. There was a distinct peak in tick density at the CSU Mountain Campus site during the week of May 27th for active surveillance, followed by a peak shortly thereafter in tick-check station density during the week of June 3rd. Notably, there was no active surveillance conducted at the CSU Mountain Campus during the weeks of June 3^rd^ or June 10th. Trends in tick density were not evaluated at the ELC and Mud Lake due to the low number of ticks collected by either method at these sites.

**Figure 5:**
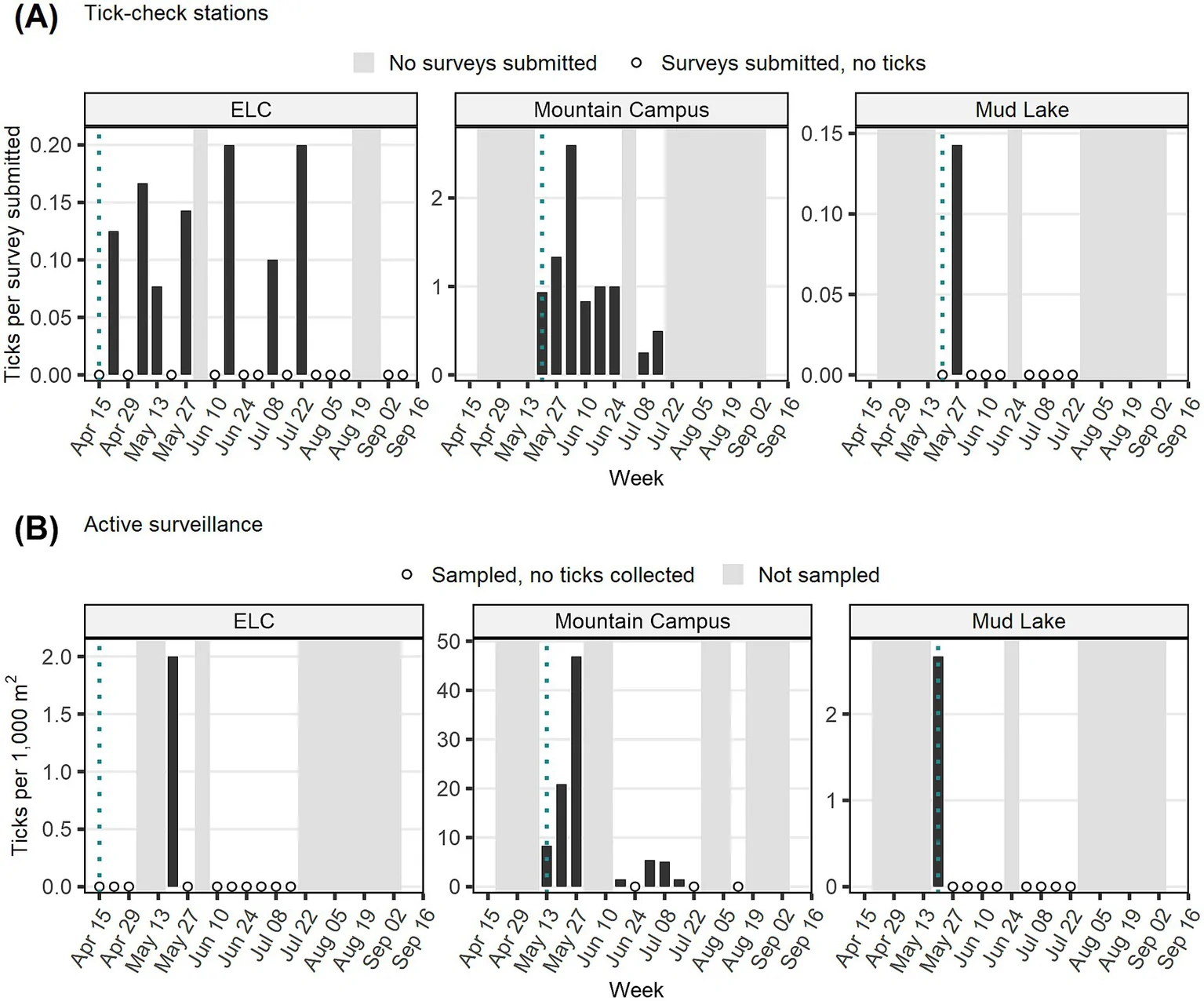
Observed tick density over time by surveillance type at three sites where ticks were collected, Colorado, April–August 2024. **(A)** Tick check-stations: density measured as ticks per survey submitted. The dotted line indicates when the tick-check station was placed on-site. Open circles on the baseline show weeks where surveys were submitted but yielded no ticks; gray bands show weeks in which no surveys were submitted, for which no density estimate exists. **(B)** Active surveillance: density measured as ticks per 1,000 m^2^ sampled. The dotted line indicates when active sampling began. Open circles on the baseline show weeks that were sampled but yielded no ticks; gray bands show weeks in which no sampling took place, for which no density estimate exists.

Next, we examined the relationship between tick densities derived from active surveillance and tick-check station yield (Figure 6). Site-periods with higher active-surveillance densities generally produced more ticks per submitted survey, although considerable variation was present among observations (Figure 6A). Because active-surveillance effort varied among site-periods, we also estimated latent active-surveillance density to account for differences in the precision of observed density estimates. Latent active-surveillance density estimates generally corresponded with observed densities, albeit with substantial uncertainty (Figure 6B). Passive tick yield increased significantly with latent active-surveillance tick density after accounting for site and the number of tick-check surveys submitted. In the Poisson mixed-effects model, latent active surveillance density was positively associated with the number of ticks obtained through passive surveillance (β = 0.378; 95% CI: 0.128–0.628; z = 2.96; *p* = 0.003). This corresponded to a 29.9% increase in expected passive tick yield for each doubling of 1 + underlying active-surveillance density (95% CI: 9.3–54.5%). Additionally, inclusion of latent active-surveillance tick density improved model fit relative to a reduced model containing survey effort and the site random intercept only (likelihood-ratio χ^2^ = 9.38; df = 1; *p* = 0.002; ΔAIC = 7.38). Simulation-based residual diagnostics showed no significant evidence of overdispersion or excess zeros (DHARMa dispersion *p* > 0.05; zero-inflation *p* > 0.05; Supplementary Figure S2). In a sensitivity analysis excluding the site-period with the highest observed active-surveillance density, the estimated association between latent density and passive tick yield remained positive but became less precise (β = 0.696; 95% CI: −0.454–1.846; *p* = 0.235).

**Figure 6:**
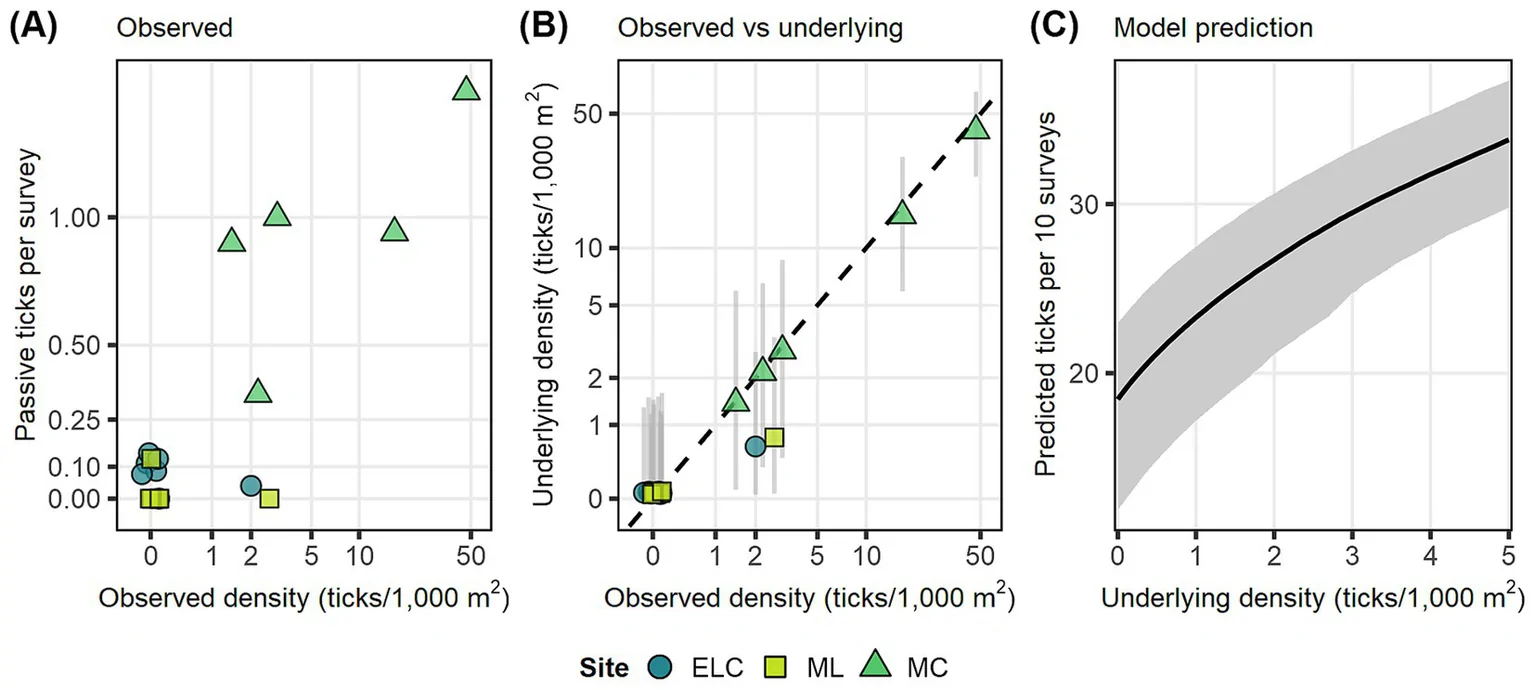
Relationship between active and passive tick surveillance. **(A)** Observed passive tick yield (ticks submitted per survey) in relation to observed active-surveillance density (ticks collected per 1,000 m sampled) for non-overlapping 14-day site-periods (*n* = 18). Points are colored and shaped by study site. **(B)** Comparison of observed and estimated underlying active-surveillance density. Underlying densities were estimated from active-surveillance tick counts and sampling effort within each site-period using a hierarchical Gamma-Poisson model. Points show posterior median estimates and vertical lines indicate 95% intervals; the dashed line represents equality between observed and underlying density. **(C)** Predicted passive tick yield per 10 surveys in relation to underlying active-surveillance density. Predictions were generated from the Poisson generalized linear mixed model with site included as a random intercept and the number of submitted surveys included as an offset. The black line shows the predicted relationship, and the shaded band represents the 95% uncertainty interval obtained by repeatedly refitting the model using values drawn from the estimated distributions of underlying active-surveillance density.

### 3.4 Tick collection effort by surveillance approach

We compared relative effort (estimated in minutes) to collect one tick by each surveillance approach (Figure 7). Sampling methods were evaluated under simulated scenarios that varied by tick density (ticks per 1,000 m^2^), one-way commute time to the site (minutes), and collection type (tick-check station, active surveillance, or a hybrid approach). The hybrid approach was defined as a combination of tick-check station and active surveillance methods. The tick-check station and hybrid approaches were further stratified by community engagement level, with scenarios representing low engagement (15 tick-check surveys submitted per month) and high engagement (30 tick-check surveys submitted per month). Effort per tick was estimated in minutes, demonstrating the relative time investment needed to collect one tick by the given collection type and scenario. Patterns of time efficiency differed across engagement levels, commute times, and tick densities (Figure 7). At low simulated tick density (0.2–1 ticks per 1,000 m^2^), both tick-station and hybrid surveillance required less effort per tick than active surveillance across all commute-time scenarios, with the greatest efficiency observed under high tick-station engagement. At four ticks per 1,000 m^2^, high-engagement tick stations and hybrid approaches generally required less effort per tick than active surveillance, whereas low-engagement tick stations were similar to or less efficient than active surveillance. Under high engagement, tick-check stations alone consistently required the least effort when sampling sites were nearby (30-min one-way commute) or when tick densities were low (0.2–1 tick per 1,000 m^2^). Under low engagement, tick-check stations required less effort than a hybrid approach only under more limited conditions, specifically at tick densities of 0.2 ticks per 1,000 m^2^, or at 1 tick per 1,000 m^2^ with a commute time of 60-min or less.

**Figure 7:**
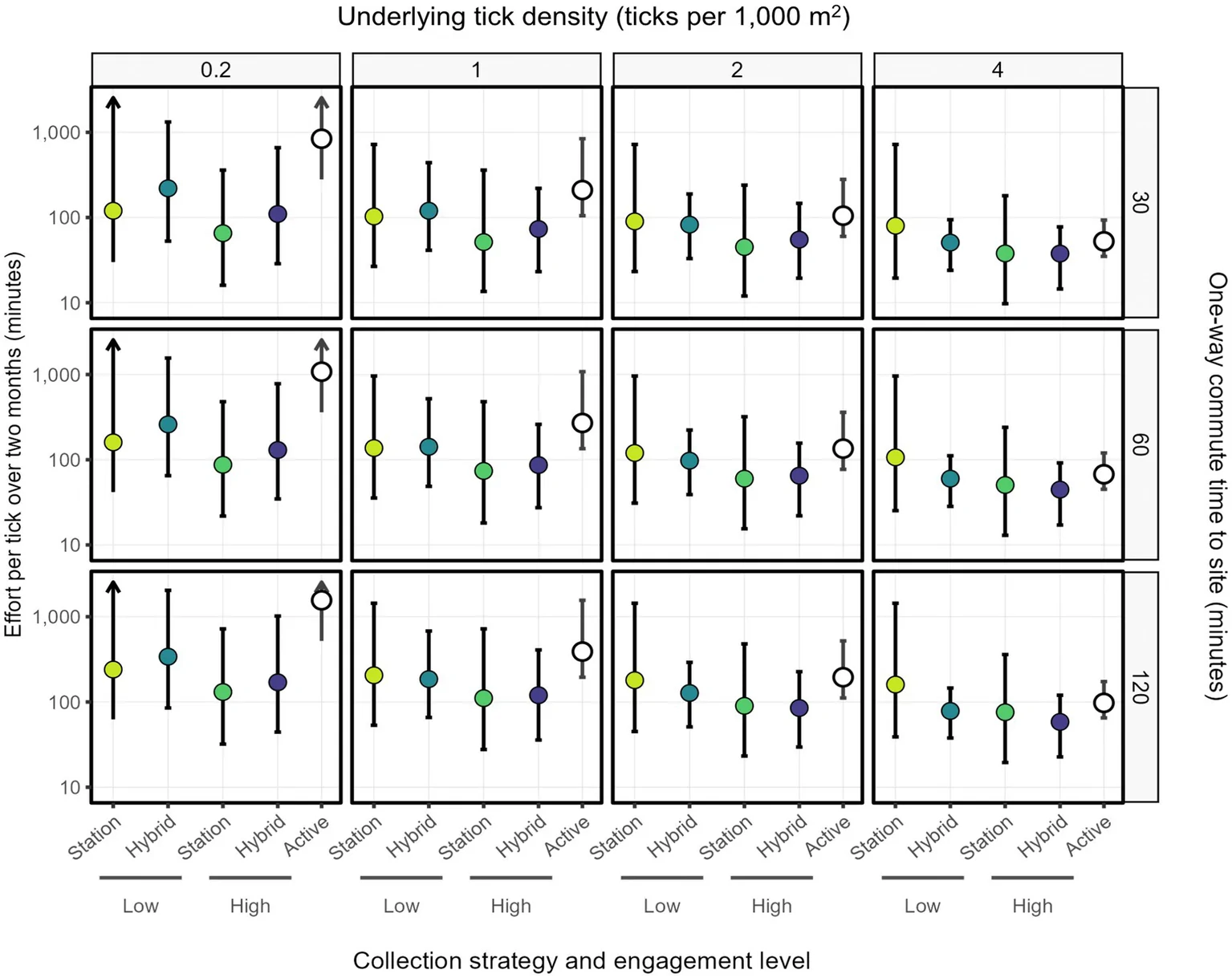
Comparison of tick-station surveillance efficiency with active surveillance and a hybrid tick-station–active sampling strategy. Surveillance efficiency was measured as total minutes of effort per tick collected over a standardized two-month surveillance period. Scenarios assumed underlying tick densities of 0.2, 1, 2, and 4 ticks per 1,000 m_2_ and one-way travel times of 30, 60, or 120 min. Tick-station and hybrid scenarios were evaluated under low (15 surveys/month) and high (30 surveys/month) passive-surveillance engagement. Active surveillance assumed two visits per month, 1,000 m_2_ sampled per visit, and 150 min of sampling effort per 1,000 m_2_. Tick stations assumed two visits per month plus 4 h/month for station preparation and data management. For hybrid surveillance, active sampling was conducted during the same site visits required to service the tick station and therefore incurred no additional travel time. Points show the median effort per tick across 5,000 simulated two-month surveillance periods, and error bars show 95% simulation intervals resulting from variation in realized tick collections. Upward arrows indicate scenarios in which the upper 95% simulation limit was unbounded because zero ticks were collected in at least 2.5% of simulated periods.

### 3.5 KAP interview results

We conducted 99 verbal KAP interviews from late June to early September 2024 (Table 2). Participants that were interviewed at sites without a tick-check station, or that reported not reading the signage posted at the tick-check station, were included in the control group (*n* = 69). Participants who reported reading the information at the tick-check station were included in the intervention group (*n* = 30).

**Table 2:** Tick and TBD knowledge of trail users interviewed at four northern Colorado trailheads, June to September 2024, by reported exposure to tick-check station signage.

Survey item	Total (*N* = 99)	Control (*N* = 69)	Intervention (*N* = 30)	Effect size (95% CI)	*p*-value
Tick-borne disease knowledge					
Disease knowledge score (Wilcoxon rank sum test)				+0.28 (rank-biserial)	0.006
Named an incorrect disease only, or named no disease	69 (70%)	54 (78%)	15 (50%)		
Named both a correct and an incorrect disease	12 (12%)	6 (8.7%)	6 (20%)		
Named a correct disease only	18 (18%)	9 (13%)	9 (30%)		
Diseases named					
Rocky Mountain spotted fever (correct)	28 (28%)	14 (20%)	14 (47%)		
Colorado tick fever (correct)	2 (2.0%)	1 (1.4%)	1 (3.3%)		
Lyme disease (incorrect)	59 (60%)	44 (64%)	15 (50%)		
Tick removal knowledge					
Described a correct element and nothing unsafe	42 (42%)	25 (36%)	17 (57%)	OR 2.28 (0.88–6.06)	0.059
Elements described					
Tweezers or forceps	43 (43%)	26 (38%)	17 (57%)		
Purpose-made tick removal device	6 (6.1%)	5 (7.2%)	1 (3.3%)		
Where to grasp the tick	17 (17%)	14 (20%)	3 (10%)		
How to pull	4 (4.0%)	2 (2.9%)	2 (6.7%)		
An unsafe method	36 (36%)	30 (43%)	6 (20%)		
Tick identification					
Identified ticks correctly from images	55 (56%)	40 (58%)	15 (50%)	OR 0.73 (0.28–1.88)	0.463

Values are *n* (%) of the column total. Three domain outcomes are tested, and *p* values are adjusted across them (Benjamini-Hochberg); the items listed beneath each domain are its components and are reported as counts only. Disease knowledge is ordered (0 = named an incorrect disease only or no disease, 1 = named both a correct and incorrect disease, 2 = named a correct disease only) and tested with a Wilcoxon rank sum test. Removal knowledge is scored as correct when a response described a tool, where to grasp the tick, or how to pull, and described nothing unsafe. Coding criteria are in Supplementary Table S3. *n* (%) of the column total. Knowledge items are compared between participants who reported reading the signage and those who did not; interviews took place as participants left the trail, so a difference is interpretable. Effect sizes are odds ratios with 95% confidence intervals from Fisher’s exact tests.

#### 3.5.1 Knowledge

Participants in the intervention group scored significantly higher in the TBD knowledge category than those in the control group (rank-biserial correlation = 0.28; *p* = 0.006). In the control group, 78% (54/69) of participants only named a non-endemic TBD, compared with 50% (15/30) in the intervention group. Conversely, 30% (9/30) of participants in the intervention group named a Colorado endemic TBD only, compared to 13% (9/69) in the control group. In the intervention group, 47% (14/30) of individuals correctly identified RMSF, compared with 20% (14/69) in the control group; only two participants across both groups correctly identified CTF as a TBD in Colorado (control *n* = 1; intervention *n* = 1). More than half of participants in both groups incorrectly identified Lyme disease as a TBD in Colorado (59/99; 60%). For tick removal knowledge, a higher proportion of participants in the intervention group described a correct removal practice and no unsafe practice (17/30, 57%) compared to the control group (25/69, 36%), although this difference was not statistically significant (OR = 2.28; 95% CI: 0.88–6.06; *p* = 0.059). There was also no significant difference in correct tick identification between groups (OR = 0.73; 95% CI: 0.28–1.88; *p* = 0.463).

#### 3.5.2 Attitudes

Overall, most participants reported *rarely*, *sometimes*, or *often* having concerns about tick bites, with fewer respondents selecting *never* or *always* (Supplementary Table S6). This distribution was consistent across both groups, and no significant differences were observed between groups (*V* = 0.13; *p* = 0.787). For concerns about TBD, overall responses were likewise concentrated in the mid-range categories (*slightly, mildly,* or *moderately concerned*), with fewer respondents selecting *not concerned* or *very concerned* (*V* = 0.24; *p* = 0.225).

#### 3.5.3 Practices

Practices related to tick bite and TBD exposure prevention were categorized as preventative behaviors (e.g., wearing protective clothing, applying repellant, and remaining on designated trails) or post-exposure behaviors (e.g., conducting a tick check, showering after outdoor activity). Across both groups, the most commonly reported preventative behavior was applying repellent (32/99, 32%), followed by wearing protective clothing (28/99, 28%) and avoiding vegetation (19/99, 19%). The most commonly reported post-exposure behavior was conducting a tick check (24/99, 24%). The proportions of participants reporting each preventive and post-exposure practice were similar between the control and intervention groups, and no statistically significant differences were observed for any individual behavior (*p* > 0.05; Supplementary Table S6; Table 3).

There was a weak positive association between disease concern and practicing any protective behavior (τ = 0.22; *p* = 0.03). The proportion of individuals reporting at least one protective behavior generally increased with higher levels of concern about TBD: 64% (7/11) among those reporting “not concerned”; 69% (18/26) “slightly concerned,” 76% (22/29) “mildly concerned,” 88% (21/24) “moderately concerned,” 100% (9/9) among those reporting “very concerned.”

**Table 3:** Relationship between concern about ticks and reporting any protective behavior, among trail users interviewed at four northern Colorado trailheads (*n* = 99).

Concern	Participants	Reported any protective behavior	Kendall’s tau (*p*)
Concern about tick bites			+0.13 (0.154; 0.154 adjusted)
Never	6	6 (100%)	
Rarely	25	16 (64%)	
Sometimes	27	20 (74%)	
Often	26	21 (81%)	
Always	15	14 (93%)	
Concern about tick-borne disease			+0.22 (0.015; 0.030 adjusted)
Not concerned	11	7 (64%)	
Slightly concerned	26	18 (69%)	
Mildly concerned	29	22 (76%)	
Moderately concerned	24	21 (88%)	
Very concerned	9	9 (100%)	

For each level of each concern scale the table gives the number of participants and the number reporting at least one protective behavior, with Kendall’s rank correlation and its *p* value adjusted across the two scales (Benjamini-Hochberg). Protective behavior means any practice listed in Supplementary Table S6. Both concern and practice are baseline characteristics, so this describes the sample rather than any effect of the signage. *n* (%) of all participants. The two right-hand columns give Kendall’s rank correlation between each reported practice and each measure of concern, with its *p* value. Attitudes and practices are baseline characteristics; the group breakdown is in Supplementary Table S7.

#### 3.5.4 Residence history

Finally, we examined associations between residence history in a high-incidence Lyme disease state with KAP outcomes (Supplementary Table S7). Participants who were current or previous residents of a high incidence Lyme disease state were significantly more likely to report engaging in a tick-bite protective behavior than other residents (effect size = 6.29; 95% CI: 1.36–59.43; *p* = 0.016). This group also had significantly higher disease knowledge scores (rank-biserial correlation = 0.28; *p* = 0.016), whereas knowledge of tick removal and identification did not differ significantly between groups.

## 4 Discussion

Given the geographical range expansion of ticks in the US and rise in reported TBD cases, effective surveillance methods are now more important than ever (1, 4, 9–11). The use of combined active tick dragging and passive tick-check stations at our study sites allowed us to evaluate the effectiveness and effort required for each method individually as well as combined via a hybrid approach. To our knowledge, this is the first systematic evaluation of tick-check stations as tools for surveillance and public education. We found that estimates of tick densities, species composition, and sex and life stage abundances derived from tick-check station data were reflective of estimates from concurrent active surveillance. Moreover, tick-check stations require less effort per tick than active surveillance under certain scenarios, particularly in areas of low tick density, when study sites are nearby, and with high station engagement. In addition to their surveillance value, the informational signage at tick-check stations may improve TBD knowledge; the value of this benefit is underscored by the low baseline TBD knowledge observed in our study. Altogether, these results suggest that incorporating tick-check stations into surveillance programs can provide reliable estimates of tick encounter risk, improve time efficiency across a range of contexts, and enhance public health education.

Passive surveillance approaches are commonly integrated into state tick surveillance programs, but their effectiveness for estimating TBD risk remains unclear. Existing evaluations of passive surveillance approaches are sparsely available and uneven in scope. One study found that distributing tick collection kits at county-level hubs resulted in the collection of more ticks than active surveillance but did not compare tick density estimates between approaches (46). Another study comparing a state mail-in system with active surveillance found that passive submissions captured county-level patterns of tick and pathogen presence comparable to active surveillance; however, no relationship was observed between estimates of tick abundance or the abundance of infected ticks across approaches (13). In our study, tick-check stations collected more ticks within Larimer County than either concurrent active surveillance or the state mail-in system. Tick-check stations also provided geographically accurate estimates of tick populations, with species composition, sex, and life stage distributions comparable to those obtained through active sampling. Moreover, spatiotemporal tick densities estimated from tick-check stations were positively associated with underlying density estimates from active surveillance after accounting for site and survey effort and potentially offering a more realistic measure of human exposure risk. Although the state’s mail-in system captured ticks across a broader geographical range, tick-check stations yielded higher tick counts, potentially increasing statistical power to detect pathogens circulating at low prevalence. Together, these findings suggest that tick-check stations represent a valuable complement to existing surveillance programs by providing spatiotemporal estimates of tick densities that scale with active surveillance and increasing sample sizes for estimating TBD risk.

Public health departments often have limited or inconsistent resources to sustain systematic tick surveillance, even as demand for tick distribution and pathogen risk information grows (5). As a result, many agencies rely on passive mail-in or voluntary tick submission systems as key components of tick surveillance activities because they are comparatively cost-effective (5, 47). However, such submission programs are typically opportunistic rather than systematic, producing data that can be spatially uneven and difficult to compare across locations or over time (14). In this context, tick-check stations may offer a useful complement to existing surveillance programs due to their efficiency and greater standardization and geographic specificity. With modest upfront investment, stations can be constructed and subsequently maintained with relatively low recurring costs. Across many scenarios, tick-check stations yielded a higher efficiency than active surveillance, particularly when tick densities were low. Moreover, implementing a hybrid model, where active dragging was conducted during routine station visits to collect submissions and replenish supplies, further reduced effort per tick across a range of scenarios. Importantly, because submissions were paired with tick-check survey data, stations also enabled assessment of encounter risk factors. We observed higher likelihood of tick encounters among individuals who reported walking through ankle-length or taller grass or brush, highlighting the value of this approach for identifying behavioral, environmental, and temporal drivers of exposure risk. Together, these findings suggest that integrating tick-check stations into existing surveillance frameworks may enhance efficiency, generate standardized estimates of tick encounter risk, and promote public engagement.

Personal protection is the primary intervention for preventing TBDs, underscoring the importance of understanding how public health campaigns lead to behavioral changes (48). In examining the knowledge component, awareness of non-Lyme TBDs was relatively low in our study, consistent with previous studies across the US (22, 49–51). More participants incorrectly identified Lyme disease as endemic to Colorado (59/99, 60%) than correctly identified RMSF (28/99, 28%) or CTF (2/99, 2%) as endemic to the state. The lack of awareness of CTF is particularly striking given that it is the most prevalent TBD in Colorado (52–54). Encouragingly, participants exposed to the tick-stations scored significantly higher on TBD knowledge than those in the control group, suggesting that this intervention may improve disease awareness. Approximately half of respondents were able to correctly describe how to safely remove a tick and identify tick images, a level of knowledge that was lower than that reported in other studies (55, 56). Collectively, these findings highlight opportunities for targeted educational interventions to address gaps in knowledge of locally relevant TBDs and suggest that tick-check stations represent one practical tool to help fill these gaps.

Within the KAP framework, attitudes encapsulates the ways which perceived risk, benefits, barriers, and personal experiences play a central role in determining whether knowledge translates into motivation for adopting protective behaviors (57–60). Consistent with this framework, we found that greater concern for TBD diseases was associated with a higher likelihood of engaging in protective behaviors. This finding aligns with previous studies in TBD contexts, which have reported that individuals with higher perceived prevalence, severity, or likelihood of contracting a TBD were more likely to engage in preventive behaviors (61, 62). At the same time, most participants in our study reported only modest concern about tick bites and TBD risk, a perception that may be proportionate to the relatively low reported incidence of TBDs in Colorado (54, 63). This level of concern differs from that reported in higher incidence regions, particularly Lyme disease-endemic areas, where a greater perceived risk of contracting a TBD is documented (57, 64). Altogether, these findings suggest that the lower perceived risk we observed in our study may be a factor in the relatively low adoption of protective behaviors reported. For example, performing a tick check is considered the gold standard for preventing tick attachment and TBD transmission (65–67); however, fewer than 25% of respondents reported performing tick checks after recreating outdoors. This value is much lower than from studies in Lyme disease endemic areas where tick-check practices often range from 45–68% (62, 64, 68). Notably, participants with a history of residence in a high incidence Lyme disease state were significantly more likely to perform protective behaviors than all other residents. This association may reflect prior experience with higher TBD risk environments, potentially including greater exposure to prevention messaging. In contrast, more participants (*n* = 32/99, 32%) reported using repellents as a tick prevention strategy; this rate is comparable to that observed in Lyme-endemic areas (62, 64, 68). The divergence between relatively low tick-check adherence and more common repellent use suggests that motivation to engage in preventative practices exists, but awareness of tick checks as an effective strategy may be limited. Although this mechanism was not explicitly assessed in this study, this pattern highlights an additional opportunity for targeted outreach and education focused on promoting awareness of evidence-based prevention strategies. Although our study design did not allow us to determine whether exposure to our tick-check station translated into sustained shifts in attitudes or practices; we established baseline measures of risk perception and preventive behaviors and identified clear opportunities to strengthen disease-specific awareness and promote behaviors that reduce TBD risk.

When utilizing tick-check stations for passive surveillance, several considerations extend beyond time and cost. Our primary goal for developing passive tick-check stations was to characterize tick-human encounters at specific trails and geographic locations. However, some submissions were inconsistent with this aim and could not be used to characterize tick exposure among trail users. For example, some individuals submitted ticks collected outside the study trails, submitted non-tick arthropods, or reported finding a tick in the tick-check survey without submitting a specimen (and vice versa). Although excluded from the final analysis, these submissions nonetheless suggest that the tick-check stations foster community engagement and interest in tick surveillance. Second, return rates for tick collection kits are another consideration. By the end of the five-month study period, 41% of distributed kits were returned; however, these kits yielded a substantial number of ticks (*n* = 80). Future implementation of similar passive collection methods should account for potential loss of supplies over time, associated replenishment costs, and sustainability challenges. Third, placement of tick-check stations should align with study objectives. If the goal is to characterize exposure across a broader landscape, stations should be placed in areas representative of where human–tick encounters are most likely to occur. Notably, the species composition of ticks submitted to CDPHE from Larimer County differed from that observed at our tick-check stations, suggesting that station placement may not have fully captured broader county-level exposure patterns. In addition, stations placed at high-traffic trailheads may maximize participation and specimen return, though they may not provide regionally representative estimates of tick community composition. Thoughtful selection of station locations is therefore essential to balance representativeness, engagement, and surveillance yield.

This study had certain limitations. No *a priori* sample-size or power calculation was performed. This was an exploratory evaluation, and the numbers of ticks collected and of KAP interview participants recruited were modest; comparisons of tick removal knowledge, tick identification, and Likert-scale attitude and practice items in particular were likely underpowered to detect small or moderate differences. Non-significant results should therefore be interpreted as inconclusive rather than as evidence that no difference exists. As a pilot study, surveillance was geographically constrained, with data collected from a limited number of trails in northern Colorado. Community engagement levels strongly influence the effectiveness of passive surveillance tools, and community participation may vary across states or regions. Outdoor recreation plays a large role in community leisure activities and culture in northern Colorado, and the recreational spaces we selected for this study were located in close proximity to multiple university campuses (69). These contextual factors may have positively influenced the level of engagement our tick-check stations received, and thus their effectiveness. Additionally, Colorado is considered to be a low tick-incidence state (54, 70). As a result, our findings may not be directly generalizable to higher incidence regions where risk perception, exposure frequency, and susceptibility to messaging fatigue or desensitization may differ. In addition, we used time as a proxy to measure effort per tick when evaluating the effectiveness of our tick-check stations under different scenarios. We did not perform a cost analysis as fluctuating prices for materials and hourly employee wages differ over time and across regions. Finally, individuals who found a tick during their tick-check may be more likely to submit a tick-check survey, limiting our ability to define a true denominator for estimating encounter rates. Future studies could address this limitation by applying Bayesian approaches to estimate the underlying denominator or by integrating trail-use metrics, such as motion sensors or traffic counters, to better approximate encounter rates. Despite these limitations, this pilot study provides important preliminary insights into the feasibility and utility of passive tick-check stations as tools for surveillance and public education.

This study compared the effectiveness of tick-check stations to traditional mail-in and active surveillance approaches. Our findings suggest that tick-check stations can engage community members in ways that enhance surveillance while simultaneously increasing TBD awareness. By leveraging community participation, this model may reduce the personnel effort typically required for active tick surveillance while generating high geographic resolution data, improving understanding of tick-human encounter risk factors, and providing community education opportunities regarding TBD risk and prevention. Overall, we conclude that tick-check stations are a promising tool that can be integrated into public health organizations’ tick surveillance programs.

## Data Availability

The raw data supporting the conclusions of this article will be made available by the authors, without undue reservation.

## Footnotes

1
https://getodk.org/
