# Supplementary material for: Comparison of tick surveillance approaches: utilizing trailhead tick-check stations to support tick surveillance and community education
Source: Front Public Health. 2026 Oct 7;14:1919913. doi: 10.3389/fpubh.2026.1919913 (PMC13683670; doi:10.3389/fpubh.2026.1919913)
Supplement: Supplementary file 1 [file Supplementary_file_1.DOCX]

Supplementary Material

Tick-check stations for community-based tick surveillance in Colorado. Generated 2026-08-28 from pipeline version 2026-08-28d.

# 1. Supplementary Methods S1. Tick-check station materials and construction

Materials, approximate costs, and step-by-step construction protocol for the tick-check stations. Prices are from summer 2024 in Colorado and are subject to change.

# 2. Supplementary Methods S2. Tick-check station survey form

Survey form supplied at each tick-check station, shown here for the Soderberg trailhead; forms at the other sites were identical apart from the site name. Respondents completed the form on returning from the trail and deposited it, with any tick found, in the station.

# 3. Supplementary Methods S3. KAP recruitment and consent script

Script used to recruit trail users for the in-person knowledge, attitudes, and practices interviews and to obtain verbal consent. Approved by the Colorado State University Institutional Review Board.

# 4. Supplementary Methods S4. KAP interview questions

Questions asked to trail users during the in-person knowledge, attitudes and practices interviews, in the order asked, with the response options offered for the closed questions. Questions 5, 6 and 8 were open-ended and were recorded verbatim; the coding applied to them is given in Supplementary Table S3. Question 7 showed participants the five specimen photographs reproduced here and asked them to point out the tick or ticks; B and D are ticks. Photographs reproduced from Cuadera *et al.* (1). Question 9 was asked only at the trailheads carrying a tick-check station.

# 5. Supplementary Figure S1. Mail-in versus tick-check station tick locations

Locations of ticks collected by passive surveillance in Larimer County, Colorado, during 2024, comparing the CDPHE mail-in system with the tick-check stations.

# 6. Supplementary Figure S2. Residual diagnostics for the passive tick yield model

Simulation-based residual diagnostics (DHARMa) for the Poisson generalized linear mixed model of passive tick yield. Left: quantile-quantile plot of scaled residuals against the uniform distribution expected under a correctly specified model, with Kolmogorov-Smirnov, dispersion and outlier tests. Right: scaled residuals against predicted values, with quantile regression lines; systematic departure from horizontal would indicate misspecification. Formal dispersion and zero-inflation test results are reported in the Methods.

# 7. Supplementary Tables

Supplementary Table S1. Characteristics of the six study sites in Larimer and Boulder counties, Colorado, at which tick-check station passive surveillance, active drag sampling, and/or in-person knowledge, attitudes and practices (KAP) interviews were conducted, April-September 2024. Coordinates are site or trailhead positions in decimal degrees (WGS 84). The expected tick species column records the a priori basis for site selection, with lower-elevation foothill sites chosen to represent Dermacentor variabilis habitat and montane sites Dermacentor andersoni habitat (2–5). Ecoregions are EPA Level III and Level IV designations, obtained by intersecting the site coordinates with the published ecoregion polygons (6). Sites lying within 5 km (Level III) or 1 km (Level IV) of a different ecoregion are labelled as a transition between the two. Vegetation is the dominant LANDFIRE (LF2025_EVT) Existing Vegetation Type within a 1000 m radius of each site coordinate, with the percentage of that buffer it occupies (7).

| **Site name** | **Location (lat, long)** | **County** | **Elevation (m)** | **Ecoregion (EPA Level III)** | **Ecoregion (Level IV)** | **Dominant vegetation type** | **Expected tick species** | **Land manager** | **Study activities** |
| --- | --- | --- | --- | --- | --- | --- | --- | --- | --- |
| CSU Environmental Learning Center (ELC) | 40.55693, -105.01921 | Larimer | 1,489 | High Plains | Front Range Fans | Northern & Central Plains Ruderal & Planted Grassland (22%) | *D. variabilis* | Colorado State University | Active sampling, tick-check station, KAP surveys |
| Soderberg | 40.53203, -105.16300 | Larimer | 1,664 | Southern Rockies / High Plains transition | Foothill Shrublands | Rocky Mountain Lower Montane-Foothill Shrubland (40%) | *D. variabilis* | Larimer County | Active sampling, tick-check station, KAP surveys |
| CSU Mountain Campus | 40.56725, -105.59400 | Larimer | 2,747 | Southern Rockies | Crystalline Subalpine Forests / Crystalline Mid-Elevation Forests transition | Rocky Mountain Lodgepole Pine Forest (51%) | *D. andersoni* | Colorado State University | Active sampling, tick-check station |
| Mud Lake | 39.97921, -105.50823 | Boulder | 2,546 | Southern Rockies | Crystalline Mid-Elevation Forests | Southern Rocky Mountain Ponderosa Pine Woodland (55%) | *D. andersoni* | Boulder County | Active sampling, tick-check station |
| Horsetooth Mountain | 40.52353, -105.18083 | Larimer | 1,764 | Southern Rockies / High Plains transition | Foothill Shrublands | Rocky Mountain Lower Montane-Foothill Shrubland (37%) | *D. variabilis* | Larimer County | KAP surveys |
| Spring Canyon trail | 40.52387, -105.16530 | Larimer | 1,659 | Southern Rockies / High Plains transition | Foothill Shrublands | Rocky Mountain Lower Montane-Foothill Shrubland (29%) | *D. variabilis* | City of Fort Collins | KAP surveys |
| Coordinates are site or trailhead positions in decimal degrees (WGS 84). The expected species column records the basis for site selection, not a result. | | | | | | | | | |

Supplementary Table S2. Demographic characteristics of KAP interview participants, for all participants and by exposure to tick-check station signage. Values are n (%) of the column total. Age was reported in fixed categories. Residence history was an open response listing every state or country a participant had lived in, so participants may contribute to more than one region, and the residence rows do not sum to the column total.

| **Survey topic** | **All participants (N=99)** | **Control (N=69)** | **Intervention (N=30)** |
| --- | --- | --- | --- |
| Age group |  |  |  |
| 18-25 | 12 (12%) | 11 (16%) | 1 (3.3%) |
| 26-35 | 22 (22%) | 19 (28%) | 3 (10%) |
| 36-45 | 15 (15%) | 7 (10%) | 8 (27%) |
| 46-55 | 20 (20%) | 13 (19%) | 7 (23%) |
| >55 | 30 (30%) | 19 (28%) | 11 (37%) |
| Residence in a high-incidence Lyme disease state | 32 (32%) | 25 (36%) | 7 (23%) |
| Current/previous residence, by census region |  |  |  |
| Midwest | 40 (40%) | 32 (46%) | 8 (27%) |
| Northeast | 17 (17%) | 12 (17%) | 5 (17%) |
| Pacific | 21 (21%) | 14 (20%) | 7 (23%) |
| South | 24 (24%) | 16 (23%) | 8 (27%) |
| Southeast | 17 (17%) | 12 (17%) | 5 (17%) |
| Southwest | 8 (8.1%) | 7 (10%) | 1 (3.3%) |
| Mountains | 6 (6.1%) | 4 (5.8%) | 2 (6.7%) |
| International | 20 (20%) | 12 (17%) | 8 (27%) |
| Outside Colorado | 87 (88%) | 62 (90%) | 25 (83%) |
| n (%) of the column total. Residence categories are not mutually exclusive, so those rows do not sum to the column total. The first column is the whole interviewed sample; the group columns partition it. | | | |

Supplementary Table S3. Questions from both the tick-check station survey form left in the drop box and the in-person interview conducted at the trailhead, with the raw response format collected, the criteria used to code each response for analysis, and the table or figure in which the results appear. One row per question; where an open-ended question was coded into several aspects, all are given in that question's row. The individual elements are listed first and any score derived from them last, so the parts are defined before the rule that combines them. Free-text responses were coded by matching pre-specified terms against the verbatim text, case-insensitively and allowing for the spelling variants present in the data.

| **Instrument** | **Survey question** | **Raw response format** | **Coding criteria** | **Results reported in** |
| --- | --- | --- | --- | --- |
| Tick-check station survey form | Current time | Time of day | Clock time, categorized as morning (<12:00), afternoon (12:00-17:00), evening (>17:00) | Table 1; Supplementary Table S5; Supplementary Table S4 |
| Tick-check station survey form | How long did you visit the trail? (hours) | Numeric, hours | Self-reported hours on trail, continuous | Table 1; Supplementary Table S5; Supplementary Table S4 |
| Tick-check station survey form | How many miles did you hike? | Numeric, miles | Self-reported miles hiked, continuous | Table 1; Supplementary Table S5; Supplementary Table S4 |
| Tick-check station survey form | Did you walk through any brush that was ankle length or higher? | Binary yes/no | Binary yes/no, walked through ankle-length or taller brush | Table 1; Supplementary Table S5; Supplementary Table S4 |
| Tick-check station survey form | What were the weather conditions like during your hike? | Select all that apply | Sunny; partly cloudy; cloudy; windy; rainy: Five independent binary indicators, one per condition, coded TRUE when that condition was selected. Each condition is tested separately. | Table 1; Supplementary Table S5; Supplementary Table S4 |
| Tick-check station survey form | Did you visit the trail with a pet? | Binary yes/no | Binary yes/no, brought a pet (prohibited at ELC and Mountain Campus) | Table 1; Supplementary Table S5; Supplementary Table S4 |
| Tick-check station survey form | Did you find a tick during your tick check? | Binary yes/no | Self-reported tick encounter: Self-reported tick encounter: Binary; the participant's own answer to whether they found a tick. Reported as a count only. | Table 1; Supplementary Table S4 |
| Tick-check station survey form | Ticks submitted with the survey form | Specimens submitted to the station | Confirmed tick encounter (derived): Confirmed tick encounter (derived): Binary; TRUE when at least one specimen submitted with the survey was confirmed to be a tick and there was no indication it had been acquired somewhere other than the trail. | Supplementary Table S5; Supplementary Table S4 |
| Tick-check station survey form | If yes, where did you find the tick(s)? | Human on clothes or skin / Dog / Other (free text) | Recorded as reported; not coded for modeling | Not reported |
| Tick-check station survey form | If yes, have you traveled out of state in the last 30 days? | Binary yes/no | Recorded as reported; not coded for modeling | Not reported |
| In-person KAP interview | Did you read the tick-check station signage? | Binary yes/no | Read signage (yes) versus did not read or no station present | Table 2 and Table 3; Supplementary Table S4 |
| In-person KAP interview | How often are you concerned about tick bites? | Five-point scale, never to always | 5-point Likert (never to always), retained as ordinal | Table 3; Supplementary Table S4 |
| In-person KAP interview | How concerned are you about tick-borne diseases? | Five-point scale, not at all to extremely | 5-point Likert (not to very concerned), retained as ordinal | Table 3; Supplementary Table S4 |
| In-person KAP interview | What tick-borne diseases occur in Colorado? | Open text | Rocky Mountain spotted fever: Open response; coded correct if Rocky Mountain spotted fever named. Colorado tick fever: Open response; coded correct if Colorado tick fever named. Lyme disease (incorrect): Open response; coded incorrect if Lyme disease named. Disease knowledge score (0-2): Correct: Rocky Mountain spotted fever, Colorado tick fever; Incorrect: Lyme, alpha-gal, ehrlichiosis, anaplasmosis; Disease knowledge score: 2 = correct only; 1 = both incorrect and correct; 0 = incorrect only or none. | Table 2; Supplementary Table S4; Supplementary Table S7 |
| In-person KAP interview | How would you remove an attached tick? | Open text | Instrument: Instrument: named tweezers, forceps or a purpose-made removal device. Grip point: Grasp point: indicated grasping at the attachment point (locative phrase required). Traction: Pulling action: described how to pull (gently, steadily, straight out, no twist). Unsafe method: Unsafe method: described any unsafe approaches (e.g. applying heat or a flame, twisting the tick, or smothering it with a substance). Removal knowledge (binary): Removal knowledge (binary): described at least one element of correct removal (tool, grasp point or pulling action) and described nothing unsafe. | Table 2; Supplementary Table S4; Supplementary Table S7 |
| In-person KAP interview | Which of these images show ticks? | Select all that apply, five images | Image-based response; correct = selected exactly the two tick images (B and D from Supplementary Methods S4) | Table 2; Supplementary Table S7; Supplementary Table S4 |
| In-person KAP interview | What precautions do you take against tick bites? | Open text | Protective clothing: Protective clothing: binary, mentions protective clothing. Repellent: Repellent: binary, mentions repellent. Avoiding vegetation: Avoiding vegetation: binary, mentions staying on the trail or avoiding tall grass. Tick check: Tick check: binary, mentions performing a tick check. Showering: Showering: binary, mentions showering. Washing clothes: Washing clothes: binary, mentions washing clothes. Pet treatment: Pet treatment: named treating a pet with an acaricide, checking a pet for ticks, or a tick-related veterinary measure. | Table 3; Supplementary Table S4 |

Supplementary Table S4. Statistical analyses behind the figures and tables of the main text, one row per test, grouped by where the result appears. Analyses reported in a supplementary table are documented there instead: tick encounter risk in Supplementary Table S5, baseline group comparison in Supplementary Table S6, and residence associations in Supplementary Table S7. Blank cells mark a quantity that does not exist for that test rather than a missing result: exact tests have no test statistic or degrees of freedom, Wald tests report z without degrees of freedom, and tests of association have no model specification. Association strength is phi for two-by-two tables and Cramer's V otherwise. Adjusted p values are given only where a family of tests was corrected together; a dash means no correction applies to that test.

| **Reported in** | **Outcome or dependent variable** | **Predictor, grouping or independent variable(s)** | **Test or model** | **Model specification** | **df** | **Effect size** | **Association strength** | **95% CI** | **P** | **Adjusted P** | **n** |
| --- | --- | --- | --- | --- | --- | --- | --- | --- | --- | --- | --- |
| Figure 3 | Surveys per week (ELC) | Week (linear trend) | Negative-binomial GLM | negative binomial (nbinom2, log link) | 1 | 0.90 (Rate ratio per week) |  | 0.85 to 0.95 | <0.001 | - | 22 |
| Figure 3 | Surveys per week (Mountain Campus) | Week (linear trend) | Negative-binomial GLM | negative binomial (nbinom1, log link) | 1 | 0.73 (Rate ratio per week) |  | 0.65 to 0.83 | <0.001 | - | 9 |
| Figure 3 | Surveys per week (Mud Lake) | Week (linear trend) | Negative-binomial GLM | negative binomial (nbinom1, log link) | 1 | 0.95 (Rate ratio per week) |  | 0.81 to 1.13 | 0.566 | - | 10 |
| Figure 3 | Surveys per week (Soderberg) | Week (linear trend) | Negative-binomial GLM | negative binomial (nbinom2, log link) | 1 | 0.90 (Rate ratio per week) |  | 0.86 to 0.95 | <0.001 | - | 18 |
| Figure 4 | Adult sex | Active vs Mail-in | Pearson's chi-squared test |  | 1 | 0.57 (Odds ratio) | -0.12 (phi) | 0.12 to 2.47 | 0.378 | 0.378 (Benjamini-Hochberg) | 53 |
| Figure 4 | Adult sex | Active vs Tick station | Pearson's chi-squared test |  | 1 | 1.65 (Odds ratio) | 0.10 (phi) | 0.57 to 5.25 | 0.309 | 0.378 (Benjamini-Hochberg) | 104 |
| Figure 4 | Adult sex | Tick station vs Mail-in | Pearson's chi-squared test |  | 1 | 0.34 (Odds ratio) | -0.20 (phi) | 0.09 to 1.05 | 0.040 | 0.119 (Benjamini-Hochberg) | 107 |
| Figure 4 | Species | Active vs Mail-in | Pearson's chi-squared test |  | 1 | 28.21 (Odds ratio) | 0.55 (phi) | 3.62 to 1300.15 | <0.001 | <0.001 (Benjamini-Hochberg) | 55 |
| Figure 4 | Species | Active vs Tick station | Fisher's exact test |  |  | 2.51 (Odds ratio) | 0.09 (phi) | 0.30 to 118.18 | 0.676 | 0.676 (Benjamini-Hochberg) | 106 |
| Figure 4 | Species | Tick station vs Mail-in | Pearson's chi-squared test |  | 1 | 11.48 (Odds ratio) | 0.49 (phi) | 3.61 to 40.55 | <0.001 | <0.001 (Benjamini-Hochberg) | 107 |
| Figure 6 | Station tick submissions | Log1p(underlying active density) | Poisson GLMM (Wald z) | poisson (log link); random: (1 \| site); offset: log(n_surveys) |  | 0.36 (log odds) |  | 0.10 to 0.63 | 0.008 | - | 18 |
| Figure 6 | Station tick submissions | Log1p(underlying active density) | Likelihood-ratio test vs intercept-only | poisson (log link); random: (1 \| site); offset: log(n_surveys) | 1 | 28.47 (% change per doubling of (1 + D)) |  | 6.87 to 54.43 | 0.004 | - | 18 |
| Figure 6 | Active tick density (ticks per 1,000 m2) | Site (partial pooling) | Hierarchical negative-binomial (Gamma-Poisson) model; estimation, no hypothesis test | nbinom2 (log link); random: (1 \| site); offset: log(exposure) |  |  |  |  |  | - | 27 |
| Table 1 | Brought a pet onto the trail | Site | Fisher's exact test |  | 3 | 0.43 (Cramer's V) |  |  | <0.001 | - | 243 |
| Table 1 | Distance hiked (miles) | Site | Kruskal-Wallis rank sum test |  | 3 | 0.24 (Epsilon-squared) |  |  | <0.001 | - | 186 |
| Table 1 | Reported finding a tick | Site | Pearson's chi-squared test |  | 3 |  | 0.80 (Cramer's V) |  | <0.001 | - | 257 |
| Table 1 | Time of submission | Site | Pearson's chi-squared test |  | 6 | 0.26 (Cramer's V) |  |  | <0.001 | - | 249 |
| Table 1 | Time on trail (hours) | Site | Kruskal-Wallis rank sum test |  | 3 | 0.08 (Epsilon-squared) |  |  | <0.001 | - | 224 |
| Table 1 | Walked through ankle-length or taller brush | Site | Pearson's chi-squared test |  | 3 | 0.27 (Cramer's V) |  |  | <0.001 | - | 254 |
| Table 1 | Weather: Cloudy | Site | Fisher's exact test |  | 3 | 0.03 (Cramer's V) |  |  | 0.972 | - | 252 |
| Table 1 | Weather: Partly Cloudy | Site | Pearson's chi-squared test |  | 3 | 0.20 (Cramer's V) |  |  | 0.021 | - | 252 |
| Table 1 | Weather: Rainy | Site | Fisher's exact test |  | 3 | 0.11 (Cramer's V) |  |  | 0.375 | - | 252 |
| Table 1 | Weather: Sunny | Site | Pearson's chi-squared test |  | 3 | 0.13 (Cramer's V) |  |  | 0.237 | - | 252 |
| Table 1 | Weather: Windy | Site | Pearson's chi-squared test |  | 3 | 0.18 (Cramer's V) |  |  | 0.046 | - | 252 |
| Table 2 | Described a correct element and nothing unsafe | Read signage | Pearson's chi-squared test |  | 1 | 2.28 (Odds ratio) | 0.19 (phi) | 0.88 to 6.06 | 0.059 | 0.088 (Benjamini-Hochberg) | 99 |
| Table 2 | Disease knowledge score (Wilcoxon rank sum test) | Read signage | Wilcoxon rank sum test |  |  | 0.28 (Rank-biserial correlation) |  |  | 0.006 | 0.018 (Benjamini-Hochberg) | 99 |
| Table 2 | Identified ticks correctly from images | Read signage | Pearson's chi-squared test |  | 1 | 0.73 (Odds ratio) | -0.07 (phi) | 0.28 to 1.88 | 0.463 | 0.463 (Benjamini-Hochberg) | 99 |
| Table 3 | Any protective behavior | Concern about tick bites | Kendall rank correlation |  |  | 0.13 (Kendall's tau) |  |  | 0.154 | - | 99 |
| Table 3 | Any protective behavior | Concern about tick-borne disease | Kendall rank correlation |  |  | 0.22 (Kendall's tau) |  |  | 0.015 | - | 99 |
| One row per test. Blank cells indicate a quantity that does not exist for that test, not a missing result: Fisher's exact tests have no statistic or degrees of freedom, Wald tests report z without degrees of freedom, and tests of association have no model specification. | | | | | | | | | | | |

Supplementary Table S5. Association between trail-use characteristics reported on tick-check station survey forms and the likelihood of finding a tick, four northern Colorado sites, April-August 2024. The outcome is whether a survey was accompanied by at least one confirmed tick: a specimen verified in the laboratory, with no indication on the survey that it had been acquired away from the trail. Two specimens submitted at Soderberg were excluded on that basis, which is why that site contributes no encounters. Crude odds ratios are from logistic regression; site-adjusted odds ratios are from binomial generalized linear mixed models with a random intercept for site, fitted separately for each characteristic. P-values for categorical characteristics are likelihood-ratio tests for the variable as a whole and are adjusted across the characteristics examined (Benjamini-Hochberg), one test per characteristic. Bringing a pet was not modeled because pets were prohibited at two sites, so the variable reflects site access policy rather than visitor behavior.

| **Characteristic** | **Surveys with a tick, n (%); or median (Q1-Q3)** | **Crude OR (95% CI)** | **Site-adjusted OR (95% CI)** | **p-value** | **p-value (adjusted)** |
| --- | --- | --- | --- | --- | --- |
| Time of submission |  |  |  | 0.215 | 0.323 |
| Morning (reference) | 12/76 (16%) | 1.00 | 1.00 |  |  |
| Afternoon | 15/108 (14%) | 0.86 (0.38-1.99) | 0.40 (0.09-1.75) | 0.223 |  |
| Evening | 25/65 (38%) | 3.33 (1.53-7.58) | 1.19 (0.28-5.01) | 0.812 |  |
| Walked through ankle-length or taller brush | 34/120 (28%) | 2.39 (1.29-4.55) | 1.17 (0.40-3.45) | 0.773 | 0.871 |
| Time on trail (per hour) | tick 1.0 (0.5-2.0); no tick 1.0 (1.0-2.0) | 1.03 (0.74-1.41) | 0.70 (0.45-1.07) | 0.100 | 0.287 |
| Distance hiked (per mile) | tick 1.4 (0.3-2.0); no tick 2.0 (1.0-3.5) | 0.72 (0.55-0.91) | 0.62 (0.43-0.87) | 0.007 | 0.011 |
| Sunny | 31/171 (18%) | 0.68 (0.36-1.29) | 0.47 (0.15-1.47) | 0.196 | 0.323 |
| Partly cloudy | 25/97 (26%) | 1.72 (0.92-3.21) | 1.07 (0.37-3.08) | 0.897 | 0.897 |
| Cloudy | 7/27 (26%) | 1.44 (0.54-3.48) | 3.96 (0.84-18.62) | 0.082 | 0.287 |
| Windy | 14/41 (34%) | 2.44 (1.15-5.05) | 2.00 (0.52-7.75) | 0.316 | 0.406 |
| Rainy | 6/17 (35%) | 2.30 (0.76-6.39) | 3.65 (0.58-23.05) | 0.169 | 0.323 |
| Site-adjusted odds ratios are from binomial generalized linear mixed models with a random intercept for site. Crude odds ratios are unadjusted. P values for categorical characteristics are likelihood-ratio tests for the variable as a whole. | | | | | |

Supplementary Table S6. Attitudes and practices by whether the participant reported reading the tick-check station signage (intervention, n = 30) or did not (control, n = 69). Values are n (%) of the column total. P values are from Pearson's chi-squared test where every expected cell count was at least five and from Fisher's exact test otherwise, and are presented to demonstrate that the two groups were comparable at baseline; they do not test an effect of the signage, since both attitudes and practices were determined before any exposure to it.

| **Survey item** | **All participants (N=99)** | **Control (N=69)** | **Intervention (N=30)** | **n compared** | **Test** | **Association strength** | **p-value (comparability)** |
| --- | --- | --- | --- | --- | --- | --- | --- |
| **ATTITUDES** |  |  |  |  |  |  |  |
| Concern about tick bites |  |  |  | 99 | Fisher's exact test | 0.13 (Cramer's V) | 0.787 |
| Never | 6 (6.1%) | 4 (5.8%) | 2 (6.7%) |  |  |  |  |
| Rarely | 25 (25%) | 19 (28%) | 6 (20%) |  |  |  |  |
| Sometimes | 27 (27%) | 20 (29%) | 7 (23%) |  |  |  |  |
| Often | 26 (26%) | 16 (23%) | 10 (33%) |  |  |  |  |
| Always | 15 (15%) | 10 (14%) | 5 (17%) |  |  |  |  |
| Concern about tick-borne disease |  |  |  | 99 | Fisher's exact test | 0.24 (Cramer's V) | 0.225 |
| Not concerned | 11 (11%) | 5 (7.2%) | 6 (20%) |  |  |  |  |
| Slightly concerned | 26 (26%) | 21 (30%) | 5 (17%) |  |  |  |  |
| Mildly concerned | 29 (29%) | 22 (32%) | 7 (23%) |  |  |  |  |
| Moderately concerned | 24 (24%) | 15 (22%) | 9 (30%) |  |  |  |  |
| Very concerned | 9 (9.1%) | 6 (8.7%) | 3 (10%) |  |  |  |  |
| **PRACTICES** |  |  |  |  |  |  |  |
| Preventive behaviors |  |  |  |  |  |  |  |
| Protective clothing | 28 (28%) | 21 (30%) | 7 (23%) | 99 | Pearson's chi-squared test | -0.07 (phi) | 0.471 |
| Repellent | 32 (32%) | 23 (33%) | 9 (30%) | 99 | Pearson's chi-squared test | -0.03 (phi) | 0.745 |
| Avoiding vegetation | 19 (19%) | 13 (19%) | 6 (20%) | 99 | Pearson's chi-squared test | +0.01 (phi) | 0.893 |
| Post-exposure behaviors |  |  |  |  |  |  |  |
| Tick check | 24 (24%) | 17 (25%) | 7 (23%) | 99 | Pearson's chi-squared test | -0.01 (phi) | 0.889 |
| Showering | 1 (1.0%) | 0 (0.0%) | 1 (3.3%) | 99 | Fisher's exact test | +0.15 (phi) | 0.303 |
| Washing clothes | 1 (1.0%) | 0 (0.0%) | 1 (3.3%) | 99 | Fisher's exact test | +0.15 (phi) | 0.303 |
| Pet-directed behaviors |  |  |  |  |  |  |  |
| Treating or checking a pet | 6 (6.1%) | 6 (8.7%) | 0 (0.0%) | 99 | Fisher's exact test | -0.17 (phi) | 0.174 |
| Any protective behavior |  |  |  |  |  |  |  |
| Any protective behavior | 77 (78%) | 54 (78%) | 23 (77%) | 99 | Pearson's chi-squared test | -0.02 (phi) | 0.861 |
| Any preventive behavior | 63 (64%) | 46 (67%) | 17 (57%) | 99 | Pearson's chi-squared test | -0.10 (phi) | 0.342 |
| Any post-exposure behavior | 26 (26%) | 17 (25%) | 9 (30%) | 99 | Pearson's chi-squared test | +0.06 (phi) | 0.577 |
| No protective behavior reported | 22 (22%) | 15 (22%) | 7 (23%) | 99 | Pearson's chi-squared test | +0.02 (phi) | 0.861 |
| P values test group comparability at baseline, not an effect of the signage: both attitudes and practices were determined before any exposure to it, and participants self-selected into reading the sign. | | | | | | | |

Supplementary Table S7. Tick knowledge and protective behavior among interview participants who had lived in a state the CDC classifies as high incidence for Lyme disease (n = 32), compared with all other participants (n = 67). Residence history was an open response listing every state or country a participant had lived in. Binary outcomes are given as n (%) of the group meeting that criterion. The disease knowledge score is ordered and is tested on that ordering, with the distribution across its three levels given as the rows beneath it. A participant met the criteria for removal knowledge if the response described at least one of three elements -- a tool for removal, where to grasp the tick, or how to pull it out -- and described no unsafe method. Unsafe methods were applying heat or a flame, twisting or spinning the tick, crushing or pinching it, and smothering it with petroleum jelly, nail polish, or a similar substance. Any one element counts rather than requiring that a tool be named, so the outcome does not privilege the component the signage was most likely to transfer. Binary outcomes were compared with Pearson's chi-squared test where every expected cell count was at least five and with Fisher's exact test otherwise, with odds ratios estimated by conditional maximum likelihood. The ordered score was compared with a Wilcoxon rank sum test, whose effect size is the rank-biserial correlation. Full coding for both derived scores is in Supplementary Table S3. P values are adjusted across the four outcomes (Benjamini-Hochberg).

| **Outcome** | **High-incidence state** | **All other residents** | **Test** | **Statistic (df)** | **Effect size (95% CI)** | **P** | **P (adjusted)** |
| --- | --- | --- | --- | --- | --- | --- | --- |
| Disease knowledge score |  |  | Wilcoxon rank sum test |  | +0.28 (Rank-biserial correlation) | 0.005 | 0.016 |
| 0 incorrect only, or none named | 16/32 (50%) | 53/67 (79%) |  |  |  |  |  |
| 1 both correct and incorrect | 7/32 (22%) | 5/67 (7.5%) |  |  |  |  |  |
| 2 correct only | 9/32 (28%) | 9/67 (13%) |  |  |  |  |  |
| Met criteria for removal knowledge | 17/32 (53%) | 25/67 (37%) | Pearson's chi-squared test | 2.22 (1) | 1.89 (0.74-4.88) | 0.137 | 0.182 |
| Identified ticks correctly from images | 18/32 (56%) | 37/67 (55%) | Pearson's chi-squared test | 0.01 (1) | 1.04 (0.41-2.67) | 0.923 | 0.923 |
| Reported any protective behavior | 30/32 (94%) | 47/67 (70%) | Pearson's chi-squared test | 6.98 (1) | 6.29 (1.36-59.43) | 0.008 | 0.016 |
| Regions are not mutually exclusive: a participant who has lived in more than one region contributes to each. The adjusted column applies the Benjamini-Hochberg correction across every comparison in this table. Comparisons significant before correction but not after are hypothesis-generating, not established associations. | | | | | | | |

# 9. Contents of this document

Included: Methods S1 (station construction)

Included: Methods S2 (survey form)

Included: Methods S3 (consent script)

Included: Methods S4 (interview questions)

Included: Supplementary Figure S1 (mail-in vs station map)

Included: Supplementary Figure S2 (DHARMa diagnostics)

Included: Supplementary Table S1 (site information)

Included: Supplementary Table S2 (demographic characteristics)

Included: Supplementary Table S3 (coding criteria)

Included: Supplementary Table S4 (statistical analyses)

Included: Supplementary Table S5 (tick encounter risk)

Included: Supplementary Table S6 (attitudes and practices by group)

Included: Supplementary Table S7 (associations with residence history)

# 10. Supplemental references

1.	Cuadera MKQ, Mader EM, Safi AG, Harrington LC. Knowledge, attitudes, and practices for tick bite prevention and tick control among residents of Long Island, New York, USA. Ticks Tick-Borne Dis. 2023 May 1;14(3):102124. doi:10.1016/j.ttbdis.2023.102124

2.	Freeman EA, Salkeld DJ. Surveillance of Rocky Mountain wood ticks (Dermacentor andersoni) and American dog ticks (Dermacentor variabilis) in Colorado. Ticks Tick-Borne Dis. 2022 Nov;13(6):102036. doi:10.1016/j.ttbdis.2022.102036 PubMed PMID: 36274450.

3.	Eisen L, Meyer AM, Eisen RJ. Climate-Based Model Predicting Acarological Risk of Encountering the Human-Biting Adult Life Stage of Dermacentor andersoni (Acari: Ixodidae) in a Key Habitat Type in Colorado. J Med Entomol. 2007 Jul 1;44(4):694–704. doi:10.1093/jmedent/44.4.694

4.	James AM, Burdett C, McCool MJ, Fox A, Riggs P. The geographic distribution and ecological preferences of the American dog tick, Dermacentor variabilis (Say), in the U.S.A. Med Vet Entomol. 2015 Jun;29(2):178–88. doi:10.1111/mve.12099 PubMed PMID: 25684582.

5.	Hutcheson HJ, Mertins JW, Kondratieff BC, White MM. Ticks and Tick-Borne Diseases of Colorado, Including New State Records for Argas radiatus (Ixodida: Argasidae) and Ixodes brunneus (Ixodida: Ixodidae). J Med Entomol. 2021 Mar 12;58(2):505–17. doi:10.1093/jme/tjaa232 PubMed PMID: 33164093.

6.	US EPA O. Level III and IV Ecoregions of the Continental United States [Data and Tools] [Internet]. 2015 [cited 2026 Aug 31]. Available from: https://www.epa.gov/eco-research/level-iii-and-iv-ecoregions-continental-united-states

7.	LANDFIRE. LANDFIRE: Existing Vegetation Type [Internet]. [cited 2026 Aug 31]. Available from: https://landfire.gov
